# Interaction of cellulose and nitrodopamine coated superparamagnetic iron oxide nanoparticles with alpha-lactalbumin†

**DOI:** 10.1039/c9ra09045b

**Published:** 2020-03-06

**Authors:** Fakhrossadat Mohammadi, Marzieh Moeeni, Chengnan Li, Rabah Boukherroub, Sabine Szunerits

**Affiliations:** Department of Chemistry, Institute for Advanced Studies in Basic Sciences (IASBS) 444 Prof. Sobouti Blvd., Gava Zang Zanjan 45137-66731 Iran fmohammadi@iasbs.ac.ir +98-24-33153232 +98-24-33153218; Univ. Lille, CNRS, Centrale Lille, ISEN, Univ. Valenciennes, UMR 8520-IEMN F-59000 Lille France sabine.szunerits@univ-lille1.fr +33 3 62 53 17 01 +33 3 62 53 17 25

## Abstract

In recent years, numerous studies have focused on the understanding of the interactions between proteins and nanoparticles (NPs). In this work, we focus on the interaction of bovine α-lactalbumin (BLA) with differently coated magnetic nanoparticles (MP): as formed MP, MPs stabilized with dopamine (MP_dopamine_) and nanoparticles coated with cellulose (MP_cellulose_). The influence of the coating on the nanoparticle–protein interaction is revealed by a set of different experiments. The binding affinity (*K*_A_) between BLA and these three structures was found to vary from 10^5^ M^−1^ (for MPs) to 10^11^ M^−1^ (MP_cellulose_). The orientation of BLA and the involvement of amino acid residues in the process of interaction with magnetic nanoparticles were identified by molecular docking studies. In addition, circular dichroism spectra revealed that the conformation of BLA was conserved upon interaction with the magnetic nanoparticles.

## Abstract 2

The adsorption of BLA on the SPIOns, and their non-toxic nature of the bioconjugate make these nanoparticles new model nanostructures for nanomedicine orientated applications.

## Graphical abstract

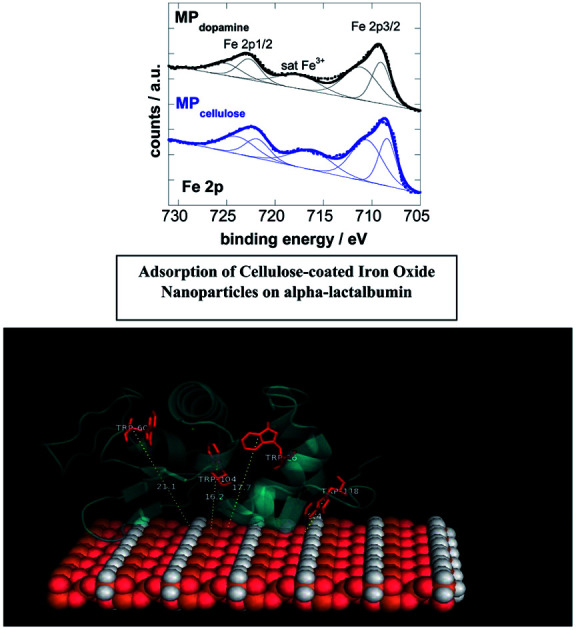

## 1. Introduction

The increased use of nanomaterials for biomedical applications necessitates an improved understanding of their behavior in biological environments. This is particularly relevant as upon injection of nanostructures into the bloodstream, nanomaterials interact with different serum proteins resulting in the formation of protein corona (PC). The structure of protein corona is a highly dynamic process with its composition changing over time and being protein dependent.^1^ Most importantly, the decoration of nanomaterials with proteins endows them with new properties which can significantly affect their biological pathways. The physicochemical properties of nanoparticles, including material composition, size, shape, surface charge, surface functionalization *etc.* have to be taken into account in the understanding of protein corona formation.^2^ A study by Tenzer *et al.* revealed that the rapid human plasma corona formation on silica and polystyrene NPs of various size and surface functionalization significantly affected nanoparticle uptake.^3^ Li *et al.* demonstrated further that protein interactions with nanoscale materials can disrupt their native conformation and compromise their biological activity.^4^ Paul *et al.* investigated the interaction of magnetic ferro fluid with bovine serum albumin. They report the key role of electrostatic interaction in the process of binding and the domain I of BSA determined as a favorable binding site.^5^ Wang *et al.* reported the reversibility and thermostability of polyethyleneimine coated silver nano particles with the two protein homologs (lysozyme and alpha lactalbumin).^6^ Carnovale *et al.* reported the impact of nanogold morphology on the interaction with human serum albumin.^7^

Most of these considerations are also valid for superparamagnetic iron oxide nanoparticles (SPIONs), which are widely used for biomedical applications.^8–14^ Hofman and co-workers investigated in this respect the effect of surface charge and coating materials of MPs on the final composition of the formed protein corona.^14^ No correlation between protein charge and nanoparticle surface charge on protein binding was observed, nor a direct correlation between the serum proteins' concentration and the proteins present.

In this work, we opted for investigating further the influence of surface coating of MPs on the interaction with bovine α-lactalbumin (BLA), a large complex of several α-lactalbumin molecules. BLA is a major component of milk proteins and possesses immunologic defense as well as antitumor and bactericidal activities. This small protein (14.2 kDa), homologues to the lysozyme family in sequence, stabilizes itself against the action of temperature and various denaturing factors upon binding the calcium cation (Ca^2+^). BLA has also been widely used as a model system as its high surface hydrophobicity has shown to be a key factor in modulating protein-nanoparticle interactions.^15,16^ Indeed, the magnetic nature of MPs and the capability of removing them upon the application of a magnetic field make these nanocarriers of high interest for biomedical applications. One of the major roles of milk proteins such as BLA is the transport of the drugs/bioactive compounds and facilitating their functionality in delivery system. By understanding the physico-chemical properties of BLA-MP corona and its stability *in vivo* experiments the new nanoplatform will be designed which has the potent drug binding ability because of the binding sites on BLA further more to its anti-bacterial and anti-tumor effects. Lystvet *et al.* reported that the immobilization onto gold nanoparticles makes alpha-lactalbumin interaction with pure and mixed phospholipid monolayers stronger compared to the native protein. This increased bioinvasiveness of protein – nanoconstructs is potentially applicable within protein based drugs and drug delivery, as well as of fundamental interest within nanotoxicology.^17^

In this work, we show that in contrast to uncoated magnetic particles (MPs) and nitrodopamine stabilized ones (MP_dopamine_), cellulose coated magnetic nanoparticles (MP_cellulose_) show high affinity for α-lactalbumin with a *K*_A_ = to 10^11^ M^−1^. The secondary structure of BLA remains unchanged upon interaction with MP_cellulose_. Docking studies were performed and identified tryptophan residues, especially Trp118, to be in closest contact with MP_cellulose_.

## 2. Experimental section

### 2.1 Materials

α-Lactalbumin (BLA), iron(ii) chloride tetrahydrate (FeCl_2_·4H_2_O), iron(iii) chloride hexahydrate (FeCl_3_·6H_2_O), dopamine hydrochloride, ammonium hydroxide (NH_4_OH) and cellulose were purchased from Sigma-Aldrich and used as received. Dulbecco's Modified Eagle's medium (DMEM, Gibco®) supplemented with 10% fetal bovine serum (FBS, Gibco®) and 1% penicillin–streptomycin (Gibco®) were purchased from Fisher Scientific. 2-Nitrodopamine was synthesized as reported previously.^18^

### 2.2 Synthesis of different Fe_3_O_4_ nanoparticle

#### 2.2.1 Synthesis of Fe_3_O_4_ nanoparticle (MP)

In this work, magnetic particles (MP) were prepared as reported previously.^19^ FeCl_2_·4H_2_O (0.34 g, 1.7 mmol) and FeCl_3_·6H_2_O (0.95 g, 3.5 mmol) were dissolved in deaerated water (20 mL). This mixture was subsequently added to a nitrogen-protected three-necked flask under sonication. The resulting mixture was heated at 50 °C for 30 min. Then concentrated ammonium hydroxide (2 mL) was added dropwise and kept at constant temperature (50 °C) for 30 min. The system was finally cooled to room temperature and the solid product was isolated *via* a non-uniform magnetic field generated by a Nd–Fe–B permanent magnet. The resulting Fe_3_O_4_ particles were washed six times with Milli-Q water to remove unreacted chemicals and then stored in water. They were lyophilized at the end of synthesis process.

#### 2.2.2 Synthesis of 2-nitrodopamine and cellulose coated MP, (MP_dopamine_) and (MP_cellulose_)

A water dispersion of bare MP (10 mg mL^−1^, 1 mL) was mixed with 2-nitrodopamine (or cellulose) (10 mg) and sonicated for 1 h at room temperature.^19^ The nitrodopamine and cellulose modified MPs were isolated by a Nd–Fe–B permanent magnet and purified through six consecutive wash/precipitation cycles with water to ensure complete removal of unreacted 2-nitrodopamine or cellulose. The precipitate was dried in an oven at 50 °C.

### 2.3 Characterization

#### 2.3.1 Transmission electron microscopy (TEM)

Transmission electron microscopy analysis of the prepared samples was carried out by using a FEI, TECNAI G2 F20 instrument operated at an accelerated voltage of 300 kV (*C*_s_ = 0.6 mm, resolution 1.7 Å). For the TEM analysis, the sample was prepared by drop casting 10 μL of the dispersed solution of 1 mg material in 5 mL isopropyl alcohol over a carbon coated 200 mesh Cu grid. The nanoparticles drop casted Cu grid was dried and the prepared sample was used for the imaging purpose.

#### 2.3.2 Magnetic properties

Magnetic properties were determined using a MPMS-XL SQUID magnetometer. The magnetization loops *M*(*H*) were measured at 300 K by sweeping the applied magnetic field between 20 kOe and −20 kOe. Thermal variation of magnetization was measured using the zero-field-cooled (ZFC) procedure, for which the sample is first cooled down to 5 K in absence of applied magnetic field, and then magnetization is measured during the warming of the sample up to 400 K, with an applied magnetic field of 80 Oe.

#### 2.3.3 UV/Vis measurements

Absorption spectra were recorded using SAFAS UV/Vis spectrophotometer (Société Anonyme de Fabrication d'Appareillages Scientifiques, Monaco) in quartz cuvettes with an optical path of 10 mm. The wavelength range was 200–1100 nm.

#### 2.3.4 Zeta-potential measurements

Zeta potential measurements were carried out by Zetasizer Nano-ZS (Malvern Instruments Inc. Worcestershire, UK). Nanomaterials were diluted to 1 nM and measured in Milli-Q water and transferred to disposable folded capillary zeta cell for zeta potential measurement.

### 2.4 Fluorescence spectroscopy

The intrinsic fluorescence spectra of BLA were recorded by Varian Cary Eclipse fluorescence spectrophotometer. The excitation wavelength was set at 280 nm and the emission spectra were recorded in the range of 300–450 nm using 10 mm quartz cells. The width of the excitation and emission slits was adjusted at 5 nm. 1 mL of 2.5 μM BLA was titrated sequentially by addition of magnetic particle solutions using a micropipet. The simultaneously scan of the excitation and emission monochromators was done for recording the synchronous fluorescence spectra. The wavelength interval (Δ*λ* = *λ*_em_ − *λ*_ex_) was fixed individually at 15 and 60 nm, at which the characteristic information of tyrosine and tryptophan residues were obtained, respectively. The Trp fluorescence intensity of the solution containing 2.5 μm BLA and 1 mg mL^−1^ magnetic particle was recorded after increasing the 5–20 mM of NaCl at the excitation wavelength of 295 nm. For FRET investigation, emission and absorption spectrum of the solution containing 1.5 μM protein and 400 μL magnetic particle (0.5 mg mL^−1^) was recorded.

### 2.5 Circular dichroism spectroscopy

Aviv spectropolarimeter model 215 (proterion Corp., USA) was used for recording the circular dichroism spectra by using 1 mm path length cell. In the far-UV region (190–260) the alteration in the secondary structure of the protein in the absence and presence of magnetic nanoparticles can be investigated. CDNN software, as an artificial intelligence program based on the neural network, was used to estimate the secondary structure content of the protein. The results were expressed in molar ellipticity (deg cm^2^ dmol^−1^).

### 2.6 Fourier transform infrared (FTIR)

FTIR spectra were recorded using a ThermoScientific FTIR instrument (Nicolet 8700) in the 550–4000 cm^−1^ range at a spectral resolution of 6 cm^−1^. 1 mg of dried sample was mixed with 200 mg of KBr powder in an agar mortar. The mixture was pressed into a pellet under 7 tons of load for 2–4 min, and the spectrum was recorded immediately. A total of 64 accumulative scans were collected. The signal from a pure KBr pellet was subtracted as a background.

### 2.7 X-ray photoelectron spectroscopy (XPS)

XPS was recorded using ESCALAB 220 XL spectrometer from Vacuum Generators featuring a monochromatic Al Kα X-ray source (1486.6 eV) and a spherical energy analyzer operated in the CAE (constant analyzer energy) mode (CAE = 100 eV for survey spectra and CAE = 40 eV for high-resolution spectra), using the electromagnetic lens mode. The angle between the incident X-rays and the analyzer is 58° and the detection angle of the photoelectrons is 30°. For XPS analysis, a small drop (∼20 μL) of the final product was placed onto a clean titanium coated substrate and allowed to dry in a vacuum desiccator. This step was repeated until a complete layer of MPs was formed on the substrate and the substrate Ti signal was minimized during XPS analysis. The samples were stored in Petri-dishes backfilled with nitrogen gas and wrapped with parafilm.

### 2.8 Theoretical investigation

Molecular docking was carried out using software Hex.8.8. Hex is an interactive molecular graphics program for calculating and displaying possible docking modes of protein. Ligands and protein were introduced to system, in PDB format. The crystal structure of BLA was downloaded from the website of protein data bank (PDB). As the size of protein is significantly smaller than nanoparticles, we assume all types of magnetic nanoparticle as a large crystalline surface. For that purpose, one layered comprising 1044 atoms each was built from the Gauss View software and the charge on the nanoparticles was kept zero.^20,21^ For instruction of the cellulose/nitrodopamine coated MP, the cellulose/nitrodopamine molecules were covered on the surface of Fe_3_O_4_ lattice by Discovery studio visualizer (DSV). The resultant capped structure of magnetic nanoparticles was then saved in PDB format for using in molecular docking simulations. Hex performs protein docking using Spherical Polar Fourier Correlations. The parameters used for docking include: correlation type – shape only, FFT mode – 3D, grid dimension – 0.6, receptor range – 180, ligand range – 180, twist range – 360, distance range – 40. Discovery studio visualizer (DSV) was used for visualizing the docked structures.^20,21^ Hex sorts the generated orientation by docking energy and prints a summary of the 10 000 highest scoring (lowest energy) orientations. The best 500 orientations are retained for viewing. The distances between bound ligands and the tryptophan residues were calculated with PyMol method.

### 2.9 Cell viability assay

The NG108-15 cell line, formed by fusing mouse N18TG2 neuroblastoma cells with rat C6-BU-1 glioma cells [ATCC® HB-12317™, ECACC, Sigma Aldrich, Saint-Quentin Fallavier, France], was cultured and maintained in Dulbecco's Modified Eagle's medium (DMEM, Gibco®) supplemented with 10% fetal bovine serum (FBS, Gibco®) and 1% penicillin–streptomycin (Gibco®) in a humidified incubator at 37 °C and 5% CO_2_. Cells were seeded at a density of 10^4^ cells per well in a 96-well plate and grown for 24 h before assay. The culture medium was replaced with a fresh medium that contains magnetic nanoparticles from 0 to 200 μg mL^−1^. After 24 h, the old medium was aspirated and cells were washed with PBS. The cell viability was evaluated using a resazurin cell viability assay. Briefly, 100 μL of the resazurin solution (11 μg mL^−1^) in DMEM/10% FBS were added to each well and the plate was incubated for 4 h in the humidified incubator. The fluorescence emission of each well was measured at 593 nm (20 nm bandwidth) with an excitation at 554 nm (18 nm bandwidth) using a Cytation™ 5 Cell Imaging Multi-Mode Reader (BioTek Instruments SAS, France). Each condition was replicated three times and the mean fluorescence value of non-exposed cells was taken as 100% cellular viability.

## 3. Results and discussion

### 3.1 Fabrication and characterization of different magnetic nanoparticles

Magnetic particles can be prepared by a set of different approaches including the co-precipitation reaction, flame spray pyrolysis, solvothermal/hydrothermal synthesis, micro-emulsion and high-thermal decomposition.^22–25^ The precipitation process, due to its simplicity, is one of the most widely employed route for the synthesis of MPs. The main advantages of this approach is the possibility to produce a large amount of material in a short time with a good control over shape and particle size (2–50 nm) by adjusting the pH, ionic strength and concentration of the growth solution. 2-Nitrodopamine^19^ and cellulose coated magnetic nanoparticles^26^ were produced in a two-step process. First, naked magnetic particles (MP) were prepared by the co-precipitation reaction of Fe^2+^ and Fe^3+^ in alkaline medium. These particles were post-coated with 2-nitrodopamine as well as cellulose, a hydrophilic polysaccharide, consisting of a linear chain of β(1 → 4) linked d-glucose units. 2-Nitrodopamine is considered an ideal ligand to stabilize magnetic particles binding irreversible to the magnetic nanostructures. In the case of cellulose, the multiple hydroxyl groups of cellulose interact strongly with the formed magnetic particles forming a stable nanocomposite.

**Fig. 1:**
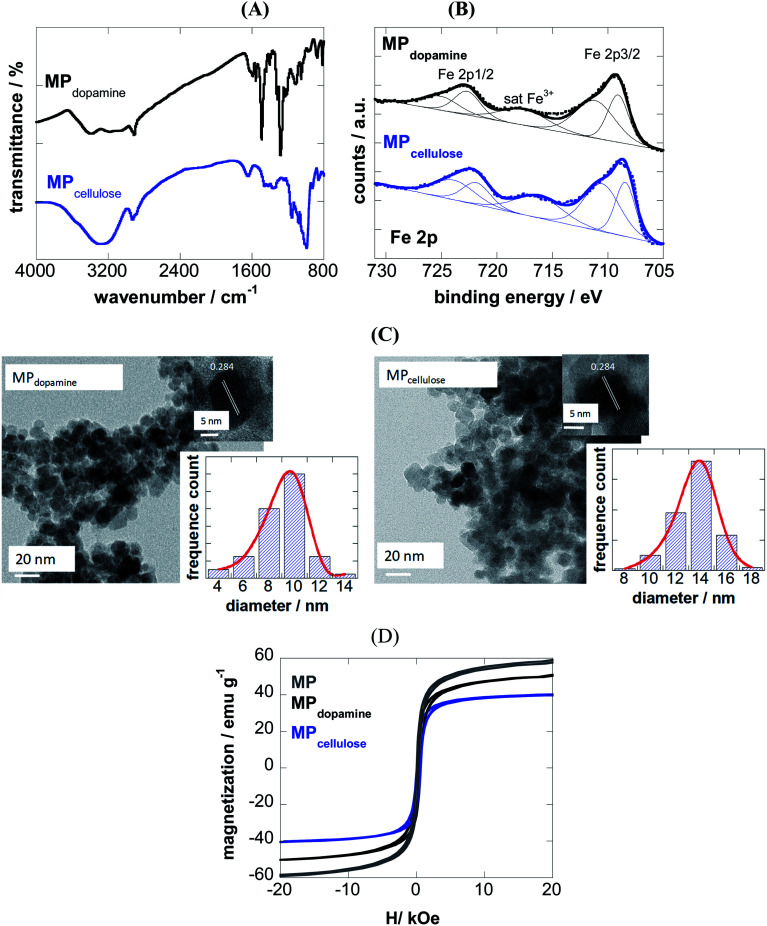
Characterization of 2-nitrodopamine and cellulose coated magnetic particles: (A) FTIR spectra; (B) Fe 2p high resolution XPS spectra; (C) Transmission electron microscopy (TEM) and HRTEM analysis, (D) zero-field-cooled (ZFC) magnetization curves with an applied field of 80 Oe and hysteresis curves at 300 K for naked MP (grey), MP_dopamine_ (black) and MP_cellulose_ (blue).

The success of coating of MP with 2-nitrodopamine and cellulose was confirmed by FTIR (Fig. 1A) and XPS analysis (Fig. 1B). The FTIR spectrum of MP_dopamine_ shows bands at 2882 and 2920 cm^−1^ (CH stretching vibrations of the dopamine ligand), ≈1290 cm^−1^ (C–O or NO_2_ vibration), and 1500 cm^−1^ (C=C vibration of the catechol system), which overlap with the asymmetric vibrations of NO_2_ at about 1548 cm^−1^. The broad band at 3367 cm^−1^ and the band at 1650 cm^−1^ are ascribed to the stretching and bending mode of the primary amines of the dopamine ligand. The FTIR spectrum of MP_cellulose_ show a large broad band centered at ≈ 3400 cm^−1^ (OH stretching of cellulose), as well as bands at 2929 cm^−1^ (C–H stretching), 1623 cm^−1^ (OH bending) and 1152 cm^−1^ (C–O–C stretching).

The survey XPS spectra of both particles show contributions of Fe 2p, O 1s, C 1s and N 1s in accordance with the chemical composition of the particles (Table 1). The density of the NH_2_ groups incorporated onto MP_dopamine_ was determined using the Kaiser test,^27^ and was estimated to be 41 ± 11 nM g^−1^, in accordance with other amino-functional magnetic particles.^28^

**Table 1:** Physico-chemical characteristics of the magnetic particles

Particles	C 1s	O 1s	N 1s	Fe 2p	Zeta potential/mV	Size/nm
MP_dopamine_	24.2	59.3	3.3	13.2	+20.2 ± 2.5	8 ± 2
MP_cellulose_	19.6	65.3	—	15.2	−22.9 ± 2.3	14 ± 3

From the Fe 2p core level XPS spectra (Fig. 1B), the bands at 709.0 and 711.0 eV indicate the presence of both Fe^2+^ and Fe^3+^ species, with satellite peak at ≈717 eV being a direct evidence for the presence of Fe^3+^ in the form of γ-Fe_2_O_3_, due to a partial oxidation of the coated magnetic particles. The degree of oxidation is comparable for both particles.

From transmission electron microscopy (TEM) images, the size of the MP_dopamine_ and MP_cellulose_ particles was estimated being 8 ± 2 and 14 ± 3 nm in diameter, respectively (Fig. 1C). The HRTEM analysis of the particles underlines the crystalline nature of the particle with a fringe width of 0.284 nm corresponding to Fe_3_O_4_ nanoparticles.

From the *M*(*H*) loops (Fig. 1D) the magnetization at 20 kOe is determined as 60 emu g^−1^ for uncoated MP lower than the saturation value (*M*_s_) reported for bulk Fe_3_O_4_ (≈92 emu g^−1^), resulting from the small particle size and large surface-to-volume ratio and other surface effects. Saturation of magnetization is obtained in the case of MP_dopamine_ and MP_cellulose_ particles, with a saturation magnetization value of 42 and 8 emu g^−1^, respectively. This reduced saturation value is related to the mass of the coating layer and possibly, the electron exchange between the surface Fe atoms and the ligands of the polymer.

### 3.2 Binding affinity of magnetic particles with BLA

The MP_cellulose_ and MP are negatively charged (*ζ*_MP_cellulose__ = −22.9 mV, *ζ*_MP_ = −22.5 mV). Upon incubation of 1 nM MP_cellulose_ and MP particles for 150 min in 5 μM BLA the zeta potential shifted to *ζ* = −32.2 mV due to the existence of highly negatively charged BLA on the MP and MP_cellulose_ surface, the first evidence for the interaction of BLA with the particles. Also the positive surface charge of MP_dopamine_ alters to negative charge (*ζ* = −22.9 mV) which confirms the firmly stick of negative BLA on the surface of MP_dopamine_ particle.

**Fig. 2:**
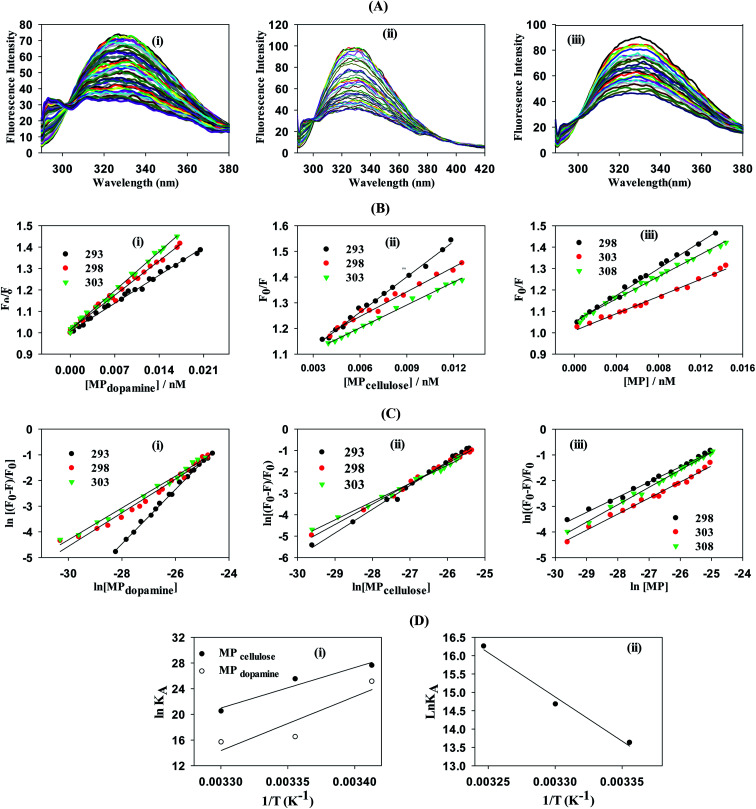
Determination of interaction strength between BLA and different magnetic particles: MP, MP_dopamine_, MP_cellulose_: (A) fluorescence quenching of BLA (2.5 μM) by (i) MP_dopamine_, (ii) MP_cellulose_ and (iii) naked magnetic particles (MP) upon excitation at *λ*_ex_ = 280 nm in the absence and presence of increasing concentration of particles (0–0.015 nM); (B) the Stern–Volmer plots for (i) MP_dopamine_, (ii) MP_cellulose_ and (iii) naked magnetic particles at different temperatures; (C) plots of ln(*F*_0_ − *F*/*F*) *versus* [particles] of BLA quenched by (i) MP_dopamine_, (ii) MP_cellulose_ and (iii) magnetic particles; (D) van't Hoff plot for (i) MP_dopamine_ and MP_cellulose_, (ii) naked magnetic particles.

To gain a better understanding on the nature and strength of the interaction taking place between BLA and the magnetic nanostructures, steady-state fluorescence measurements were performed.^20^ BLA contains four tryptophan residues which are responsible for its intrinsic fluorescence with tyrosine and phenylalanine units contributing in addition to the fluorescence signal. Upon excitation at 280 nm, BLA displays an emission at 338 nm, which is quenched in the presence of increasing concentrations of MP, MP_dopamine_ and MP_cellulose_ (Fig. 2A). Analysis of the fluorescence data using the Stern–Volmer equation,1
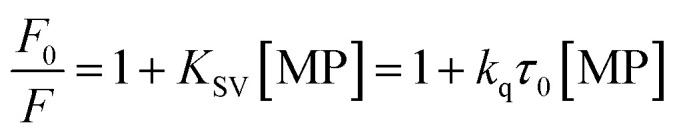
where *F*_0_ and *F* are the fluorescence intensities in the absence and presence of particles, *K*_SV_ the Stern–Volmer quenching constant, *k*_q_ the bimolecular quenching rate constant, and *τ*_0_ the average lifetime of the biomolecule without the quencher, respectively, allows determination of the binding constant *K*_A_ between BLA and the magnetic particle using eqn (2) with *n* being the Hill coefficient.2
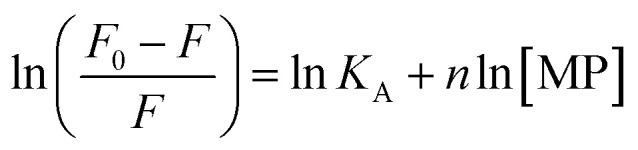


From a plot of *F*_0_/*F versus* [MP] (Fig. 2B), the Stern–Volmer constant can be determined from the slope (Table 2). The value of *K*_SV_ is decreasing for MP_cellulose_, while increasing for MP_dopamine_. Assuming a decay time of *τ*_0_ = 10^−8^ s, a *k*_q_ in the order of 10^19^ M^−1^ s^−1^ is obtained which is greater than the diffusion-controlled quenching value of 10^10^ M^−1^ s^−1^.^29^ This confirms that the quenching initiated with MP, MP_dopamine_ and MP_cellulose_ is not diffusion controlled and the result of binding interaction between BLA with the magnetic nanostructures. A plot of ln(*F*_0_ − *F*/*F*) (eqn (2)) (Fig. 2C) allowed the determination of *K*_A_ (Table 2). Compared to other reported binding constants of proteins to particles, which are in the order of 10^6^ to 10^8^ M^−1^,^13,30,31^ (Table 3), the binding constant of BLA to MP_cellulose_ is strong. Its value is decreasing with increasing temperature due to unreduced adsorption of the protein to the particles.^32,33^ This indicates a static quenching mechanism and decrease in the quantum yield caused by the formation of a non-fluorescent ground state complex.^34^ This is in contrast to naked MP where the value of *K*_A_ is increasing with increasing temperature which refers to dynamic quenching mechanism caused by the collisional encounter between the fluorophore and quencher molecules.^34^

**Table 2:** Change in Stern–Volmer quenching constant (*K*_SV_), bimolecular quenching rate constant (*k*_q_), the number of binding sites *n*, and binding constant (*K*_A_) between magnetic particles and BLA = 2.5 μM at different temperatures

Particle	*T*/K	*K*_SV_/M^−1^	*k*_q_/M^−1^ s^−1^	*n*	*K*_A_/M^−1^
MP_cellulose_	293	(4.50 ± 0.04) × 10^10^	(4.50 ± 0.04) × 10^19^	1.12 ± 0.02	(9.68 ± 5.01) × 10^11^
	298	(3.64 ± 0.07) × 10^10^	(3.64 ± 0.07) × 10^19^	1.04 ± 0.02	(1.18 ± 0.70) × 10^11^
	303	(3.60 ± 0.06) × 10^10^	(3.60 ± 0.06) × 10^19^	0.85 ± 0.02	(7.91 ± 3.62) × 10^8^
MP_dopamine_	293	(1.84 ± 0.03) × 10^10^	(1.84 ± 0.03) × 10^19^	1.05 ± 0.02	(8.03 ± 4.39) × 10^10^
	298	(2.29 ± 0.02) × 10^10^	(2.29 ± 0.02) × 10^19^	0.70 ± 0.02	(1.43 ± 0.73) × 10^7^
	303	(2.62 ± 0.03) × 10^10^	(2.62 ± 0.03) × 10^19^	0.67 ± 0.02	(6.41 ± 3.80) × 10^6^
MP	298	(3.01 ± 0.07) × 10^10^	(3.01 ± 0.07) × 10^19^	0.58 ± 0.02	(8.29 ± 3.81) × 10^5^
	303	(1.97 ± 0.05) × 10^10^	(1.97 ± 0.05) × 10^19^	0.64 ± 0.03	(2.01 ± 1.50) × 10^6^
	308	(2.71 ± 0.07) × 10^10^	(2.71 ± 0.07) × 10^19^	0.69 ± 0.01	(1.14 ± 3.33) × 10^7^

**Table 3:** Binding constants of different proteins to magnetic nanostructures

Protein	MP	*K*_A_/M^−1^	Ref.
Bovine serum albumin	Fe_3_O_4_ nanoparticles stabilized by SDBS	2.40 × 10^8^	30
Immunoglobulin	Fe_3_O_4_ nanoparticles stabilized by PEG	2.61 × 10^6^	13
Bovine serum albumin	Dendrimer coated magnetite nanoparticles	21.1 × 10^8^	31

The *n* value decreases in addition with higher temperature due to the decrease of the cooperativity due to conformal change and/or further protein adsorption.

Finally, using eqn (3) and (4)3
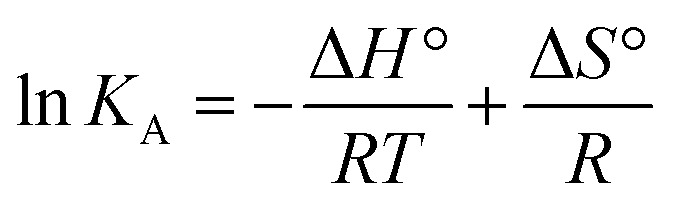

4 Δ*G*° = Δ*H*° − *T*Δ*S*°

with *R* being the universal gas constant, the thermodynamic parameters (Δ*H*° and (Δ*S*°) as well as the Gibbs free energy (Δ*G*°) were determined (Table 4). A meaningful relationship between the sign of the thermodynamic parameters and the types of interaction that contribute in ligand–protein binding process was confirmed by Ross and Subramanian in 1981.^35^ According to the enthalpy and entropy changes in the case of MP_cellulose_ and MP_dopamine_ with Δ*H*° < 0 and Δ*S*° < 0, the modes of interaction between BLA and the particles are van der Waals interactions and hydrogen bonds, occurring spontaneously (Δ*G*° < 0). This is in contrast to MP, where hydrophobic forces are believed to be mainly responsible for the interaction of BLA with MPs.

**Table 4:** Thermodynamic parameters of the interaction of magnetic nanoparticles with BLA (2.5 μM) at 298 K

Particle	Δ*H*°/kJ mol^−1^	Δ*S*°/J mol^−1^ K^−1^	Δ*G*°/kJ mol^−1^
MP_cellulose_	−124 ± 1.1	−369 ± 3.8	−14 ± 4.0
MP_dopamine_	−166 ± 1.2	−521 ± 3.2	−11 ± 4.5
MP	47 ± 1.0	186 ± 2.9	−8 ± 3.9

#### 3.2.1 Adsorption isotherms of BLA to MP_cellulose_

The high binding constant of MP_cellulose_ might be related to the good capacity of the nanoparticles to adsorb BLA. Analyzing the amount of BLA in the solution before and after adsorption at 289 nm using UV/Vis measurements, allowed determining the adsorption capacity [*q*_e_ (mg g^−1^)] using eqn (5)5
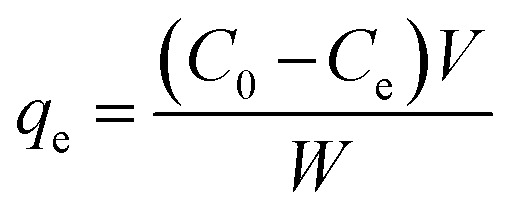
where *C*_0_ (mg mL^−1^) and *C*_e_ (mg mL^−1^) are the initial and final BLA concentrations, *V* (mL) the volume of the solution and *W* (g) the weight of adsorbents (magnetic particles).

Assuming the formation of an adsorbate monolayer on the particles, application of the Langmuir adsorption isotherm (eqn (6))6
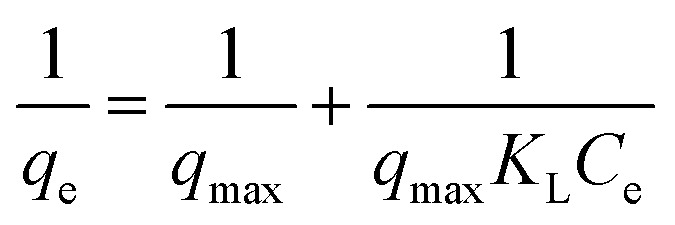
where *q*_max_ is maximum monolayer coverage capacity (mg g^−1^), *C*_e_ (mg mL^−1^) the equilibrium concentration of BLA in the solution and *K*_L_ (mL mg^−1^) is the Langmuir constant, allowed determining *q*_max_ and *K*_L_ (see ESI, Fig. S1†) from the slope and intercept of the Langmuir plot (Table 5).

**Table 5:** Langmuir and Freundlich isotherm constants for adsorption of BLA on magnetic nanoparticles as determined from Fig. S1

Particles	Isotherm	Parameters	Values
MP_cellulose_	Langmuir	*q*_max_ (mg g^−1^)	833 ± 23.31
		*K*_L_ (mL mg^−1^)	12 ± 1.04
		*R*_L_	0.48 ± 0.01
		*R* ^2^	0.99
	Freundlich	*K*_f_ (mL g^−1^)	728 ± 21
		1/*n*	0.22 ± 0.02
		*R* ^2^	0.98
MP_dopamine_	Langmuir	*q*_max_ (mg g^−1^)	714 ± 13.12
		*K*_L_ (mL mg^−1^)	14 ± 0.96
		*R*_L_	0.15 ± 0.03
		*R* ^2^	0.99
	Freundlich	*K*_f_ (mL g^−1^)	658 ± 35
		1/*n*	0.18 ± 0.01
		*R* ^2^	0.97
MP	Langmuir	*q*_max_ (mg g^−1^)	666 ± 7.51
		*K*_L_ (mL mg^−1^)	7.5 ± 0.21
		*R*_L_	0.17 ± 0.02
		*R* ^2^	0.99
	Freundlich	*K*_f_ (mL g^−1^)	577 ± 23
		1/*n*	0.19 ± 0.02
		*R* ^2^	0.93

A plot of 1/*q*_e_*vs.* 1/*C*_e_ allowed determining the equilibrium parameter *R*_L_ (eqn (7)) a dimensionless constant referred to as separation factor or equilibrium parameter:7
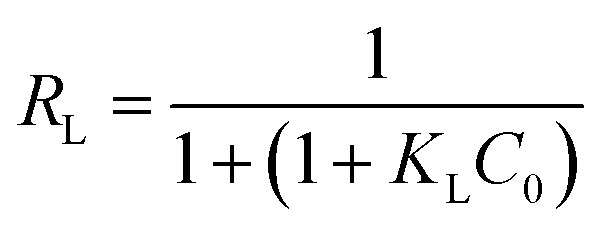


*R*_L_ > 1 indicates unfavorable adsorption, 0 < *R*_L_ < 1 favorable and *R*_L_ = 0 irreversible adsorption. The determined *R*_L_ values are greater than 0 but less than 1, indicating that under the Langmuir isotherm model adsorption of BLA onto the particles is favorable. The first assumption of the Langmuir model is monolayer coverage of the adsorbed molecules on the surface. Generally, there is no tendency for protein molecules to adhere to one another and this causes the nanoparticles surface to be saturated with monolayer coverage of protein molecules. Another important assumption of the Langmuir model is non-interacting adsorbent species on the surface. The CD measurements and synchronous fluorescence results provide evidences that the secondary and tertiary structures of the protein were conserved and aggregation or clustering among the adsorbed BLA molecules on the nanoparticles surface did not take place; hence this assumption of the Langmuir model is satisfied. Though the current results are well fitted to the Langmuir isotherm, but sometimes all the required conditions of this isotherm model for adsorption of proteins on the surface are not satisfied, so it is better to suggest that adsorption data have Langmuir-looking behavior.^36^

#### 3.2.2 Freundlich adsorption isotherm

This model used to describe the adsorption characteristics for the heterogeneous surface which considers multilayer adsorption. The linear form of Freundlich equation is represented by the following equation:
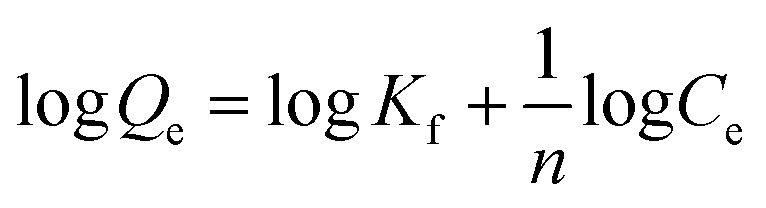
where *K*_f_ (mL g^−1^) is the Freundlich adsorption isotherm constant relating to the extent of adsorption and 1/*n* is related to the strength of adsorption in the adsorption process, which varies with the heterogeneity of the material. The slope and intercept of the Freundlich plot of log *q*_e_*vs.* log *C*_e_ was used for obtaining the 1/*n* and *K*_f_ values (see ESI, Fig. S1†). If the value of 1/*n* is below one, it indicates a normal adsorption. On the other hand, 1/*n* being above one indicates cooperative adsorption. The extracted Freundlich parameters are listed in Table 5. As can be seen, the Langmuir-looking model has a slightly better description for the adsorption of BLA molecules on the surface of magnetic particles.

#### 3.2.3 Electrostatic contributions

Salt titration experiments were performed in addition to evaluate the contribution of electrostatic interactions between magnetic nanoparticles and BLA in the process of adsorption. Magnetic particle/protein solutions were titrated with sodium chloride and the release of BLA through disruption of the electrostatic interaction and recovery of the Trp fluorescence were determined (see ESI, Fig. S2†). In the case of MP_cellulose_ and MP_dopamine_ the recovery of the Trp fluorescence intensity upon increasing the ionic strength was observed, indicating the presence of electrostatic interactions. So the more adsorptive capacity in the MP_cellulose_ and MP_dopamine_ cases are reasonable due to the contribution of both electrostatic interaction and hydrogen bonding.

### 3.3 Energy transfer from the BLA to MPs

**Fig. 3:**
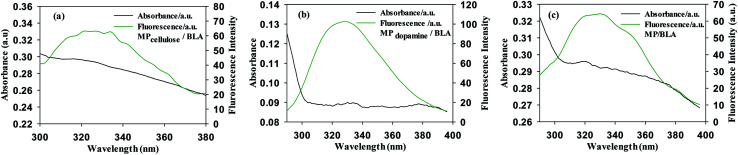
Emission and absorption spectra of (a) MP_cellulose_/BLA, (b) MP_dopamine_/BLA and (c) MP/BLA, *λ*_ex_ = 280 nm.

BLA fluorescence quenching in the presence of increasing concentrations of MP could be the result of energy transfer. Fig. 3 shows a spectral overlap between the emission spectrum of BLA and absorption spectrum of the studied magnetic particles. According to the Förster's theory (see ESI1†), parameters such as the Förster critical distance *R*_0_, *J* a factor describing the overlapping between the emission spectrum of the donor and the absorption spectrum of the acceptor (particle in our case) and the *r* value were determined (Table 6). A non-radiative energy transfer occurred with high probability between the magnetic particles and BLA because of the estimated distance values which is on the 2–7 nm scale.

**Table 6:** Förster's theory: determination of Förster critical distance *R*_0_, the distance between the donor and acceptor *r*, *J* a factor describing the overlapping between the emission spectrum of the donor and the absorption spectrum

Förster parameter	MP_cellulose_	MP_dopamine_	MP
*R*_0_ (nm)	4.5–5.5	3.7–4.5	4.5–5.4
*r* (nm)	4.2–5.1	4.6–5.5	4.9–5.9
*J*(*λ*) (M^−1^ cm^3^)	2.42 × 10^−13^	7.73 × 10^−14^	2.51 × 10^−13^
*E*	0.4099	0.2197	0.3776

### 3.4 Circular dichroism (CD)

Circular dichroism (CD) spectroscopy was in addition used to obtain information about eventual protein deactivation. As illustrated in Fig. S3,† the secondary structure of BLA remains intact upon adsorption onto the magnetic particles (Table 7).

**Table 7:** Percentage of secondary structural content of BLA in the absence and presence of magnetic particles

Secondary structure	BLA	MP_cellulose_/BLA	MP_dopamine_/BLA	MP/BLA
α-Helix	22.6	24	22.2	23.1
β-Sheet	25.2	25	26.0	25.4
β-Turn	17.3	17.6	17.5	17.5
Random coil	34.8	33.4	33.9	33.7

### 3.5 Conformational investigation by synchronous fluorescence

**Fig. 4:**
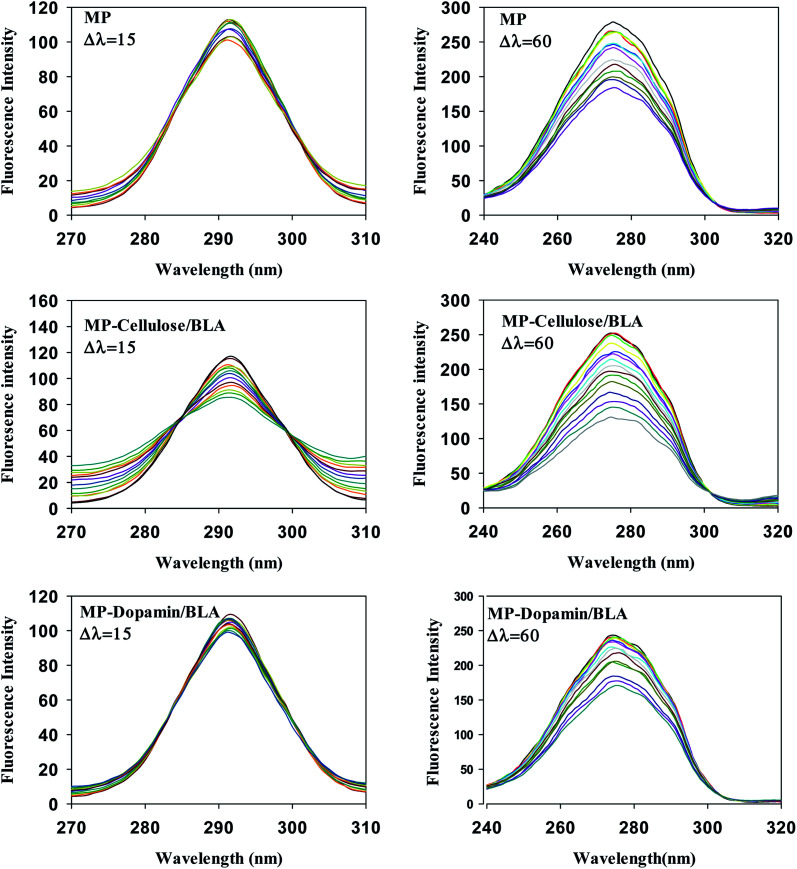
The effect of the magnetic nanoparticles on the synchronous fluorescence of BLA at Δ*λ* = 15 and Δ*λ* = 60. [BLA] = 2.5 μM, [ligands] = 0–30 μM.

The important technique which provides interesting information about the micro region of fluorophore molecules is synchronous fluorescence spectroscopy. It offers information about the molecular environment characteristics such as polarity in the vicinity of the fluorophore molecules. According to the theory of Miller, by taking synchronous spectra at the selected wavelength difference, Δ*λ*(*λ*_em_ − *λ*_ex_), the fluorescence spectral properties of tryptophan and tyrosine residues can be separated. When Δ*λ* is 15 nm the fluorescence spectrum reflects the emission by tyrosine residue whereas the difference of 60 nm between excitation and emission wavelengths gives the information about tryptophan residue.^37^Fig. 4 shows the synchronous spectra of all types of magnetic nanoparticles with BLA at Δ*λ* of 15 nm and 60 nm. The extent of the quenching in the case of Δ*λ* = 60 nm, is greater than that of Δ*λ* = 15 nm. So, the Trp residues of BLA are at close vicinity of magnetic nanoparticles. In the spectra at Δ*λ* = 60 no considerable shift in the *λ*_max_ was observed upon addition of all types of magnetic nanoparticles. So, it can be verified that the polarity of the micro region of the tryptophan residues does not change in the process of binding.

### 3.6 Cell viability

**Fig. 5:**
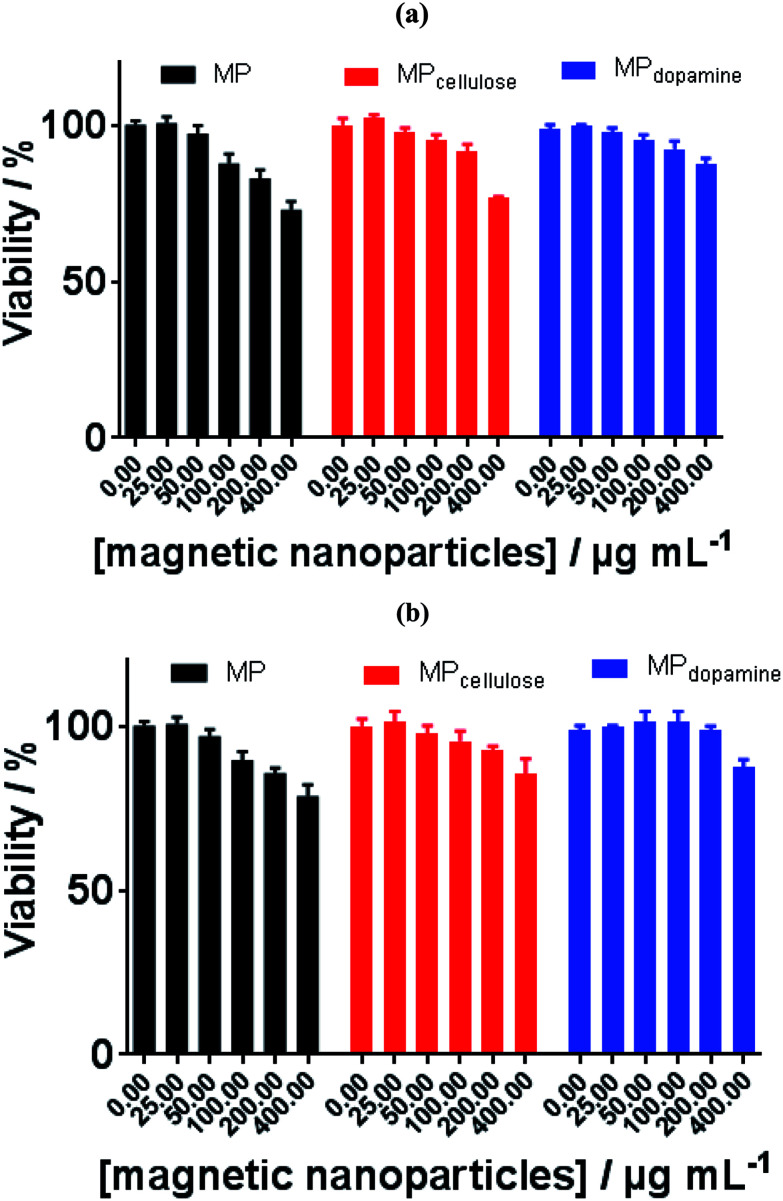
Cell viability of MP, MP_cellulose_, and MP_dopamine_ alone (a) and after incubation with BLA (b).

Toxicity has already become a significant biological issue of MPs, and thus beside the physicochemical investigation of interaction of the considered MPs in the current work with BLA, their toxicities were further studied. The cell toxicity of the MP, MP_dopamine_ and MP_cellulose_ alone and after incubation with BLA forming MP/BLA bioconjugate was established on neuroblastomaxglioma hybrid cell line, NG108-15, widely used in *in vitro* studies instead of primary-cultured neurons (Fig. 5). Cell viability was assessed by the well-established resazurin assay, which does not suffer from unspecific interaction with magnetic particles. Particles' toxicity was evaluated based on cell viability assessment relative to controls as proposed by Kong *et al.*^38^: cell viability >90% correlates with non-toxic nanoparticles, cell viability of 60–90% reveals slight toxicity, cell viability between 30–60% shows toxic features, and cell viability <30% corresponds to severe toxicity. In the absence of BLA, MP is non-toxic up to 50 μg mL^−1^, whereas MP_dopamine_ and MP_cellulose_ are non-toxic up to 200 μg ML^−1^. After bioconjugate formation with BLA, the cell viability of 100 μg ML^−1^ of MP increased to more than 90%, and for 400 μg mL^−1^ of MP_dopamine_ and MP_cellulose_, the cell viability increased to more than 80%. The results of cytotoxicity showed that not only coating of magnetic particles with cellulose and dopamine results in increasing the cell viability, but also formation of BLA corona, caused that MP_dopamine_ and MP_cellulose_ to be non-toxic even at higher concentration. The cell viability results reported so far revealed that the toxicity of bare MPs are higher than for other coated MPs, due to their tendency to adsorb proteins, amino acids, vitamins, and ions causing changes in pH and composition in cells and cell media.^39^

### 3.7 Molecular docking

**Fig. 6:**
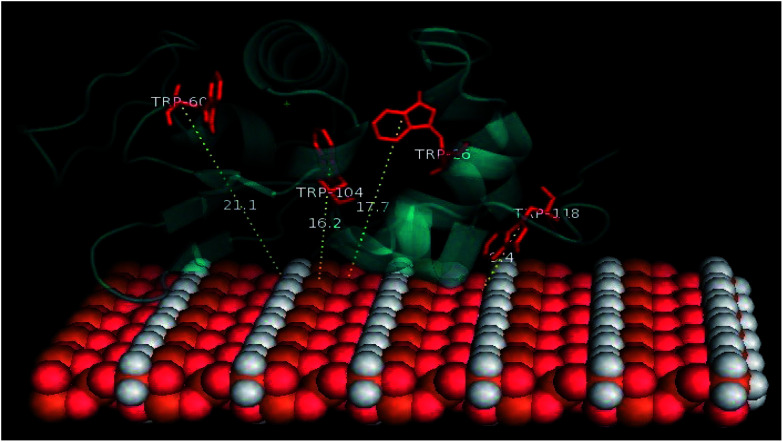
The calculated distances between MP-sheet and four tryptophans in BLA rendered by PyMol.

Docking studies were finally performed in order to identify the involvement of amino acids in the binding of BLA to the MPs. The five best docked BLA structures with MP_cellulose_, MP_dopamine_, and MP (see ESI, Fig. S4–S6†) indicate the formation of an energetically stable corona coating. The closeness of tryptophan residues, especially Trp118 to MP (Fig. 6), is in agreement with the fluorescence quenching results. There are few reports about the environment of BLA tryptophans.^40–42^ Based on these few reports Trp-118 is the most exposed tryptophan, while Trp-26 is a buried residue in the protein, harbored in a hydrophobic pocket. Trp-60 and Trp-104 are close to each other; Trp-60 is largely solvent excluded, while Trp-104 is partially solvent inaccessible. The tail conformation of Trp-118 determines its position either in a buried or exposed state. The same results were obtained by Takase *et al.* (1977)^40^ about the environment of BLA tryptophans. They suggested that Trp-26 is solvent inaccessible residues and located in the nonpolar interior of the protein matrix. Trp-60 is almost shielded from the solvent. Based on their results a cleft-like region which contains Trp-60 and either Trp-104 or Trp-118 exists in the protein molecule. Either Trp-104 or Trp-118 is largely solvent accessible to solvent or located on the molecule surface.^40–42^

It seems that Trp-118 is more likely attainable to magnetic particles because it is the most exposed tryptophan. Trp-104 is the most exposed tryptophan, so the possibility of binding the ligands to this residue also exists. Trp-26 and Trp-60 are buried in the interior of the protein and are inaccessible to the solvent. Docking studies results can help to better understanding of the details of the contribution of tryptophans in the reduction of fluorescence intensity of BLA during the binding process. The CD spectroscopy illustrated no altering in the second structure of protein in the process of binding and by the synchronous fluorescence results no polarity altering around the tryptophan residues was observed, so the hydrophobic patches of protein are not exposed to solvent and the tryptophans like Trp-26 and Trp-104 which are buried in their pocket will still be hided from the solvent. By docking investigation, the closeness of magnetic particles to Trp-118 is confirmed. It seems there is no penetration of BLA on the magnetic particles. So the protein slightly attaches to the magnetic particle and consequently forming the corona. Trp-118 has the capability of being the key chromophore which is quenched by quencher molecules called magnetic particle in this work.

The PyMol method was used for calculating the distance of four tryptophan residues (Trp-26, Trp-60, Trp-104 and Trp-118) to magnetic particles sheets. These distances are comparable with experimental distances from FRET calculation (Tables 8 and 9).

**Table 8:** The total energy and the interface amino acids of various orientations of MP_cellulose_/BLA interaction

Docked pose	Total energy (a.u.)	Amino acids
1	−668.66	Asp14, Leu15, Lys16, Gly17, Tyr18, Gly19, Gly20, Val21, Ser22, Leu23, pro24, Glu25, Lys93, Lys94, Ile95, Leu96, Asp97, Lys98, Val99, Gly103, Ile101, Asn102, Tyr103, His107, CYs111, Ser112, Leu115, Asp116, 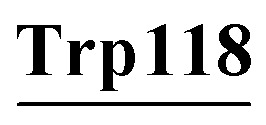 , Leu119
2	−571.76	Lys16, Gly17, Tyr18, Gly19, Gly20, Val21, Ser22, Lys93, Lys94, Ile95, Leu96, Asp97, Lys98, Val99, Gly103, Ile101, Asn102
3	−559.92	Asp14, Leu15, Lys16, Gly17, Tyr18, Gly19, Gly20, Val21, Ser22, Leu23, pro24, Glu25, Lys93, Lys94, Ile95, Tyr103, His107, CYs111, Ser112, Leu115, Asp116, 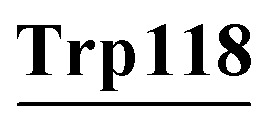 , Leu119
4	−545.77	Lys16, Gly17, Tyr18, Gly19, Gly20, Val21, Ser22, Leu23, pro24, Glu25, Lys93, Lys94, Ile95, Tyr103, His107, CYs111, Ser112, Leu115, Asp116, 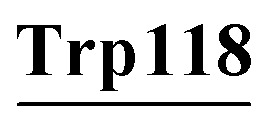
5	−538.67	pro24, Glu25, Lys93, Lys94, Ile95, Tyr103, His107, CYs111, Ser112, Leu115, Asp116, 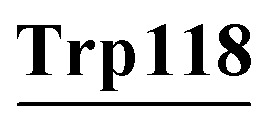 , Leu119

**Table 9:** The parameters of the five stable MP_cellulose_/BLA complex, as obtained by molecular docking. The calculated distances between the interface interactive amino acids and the MP_cellulose_-sheet and the types of bindings which contributed in the binding site

Docked pose	Amino acids	Distances (Å)	Category	Type
Pose 1	Lys16	2.36	Hydrogen bond	Conventional
	Tyr18	2.74	Hydrogen bond	Conventional
	Le115	3.03	Hydrogen bond	Conventional
	Asp14	2.35	Hydrogen bond	Conventional
	Glu25	2.73	Hydrogen bond	Conventional
	Gly19	2.66	Hydrogen bond	Conventional
	Gly19	2.84	Hydrogen bond	Carbon
	Gly19	2.16	Hydrogen bond	Carbon
	Sr112	2.38	Hydrogen bond	Carbon
	Lys16	5.18	Hydrophobic	Alkyl
	Val21	4.61	Hydrophobic	Alkyl
	Ile101	4.31	Hydrophobic	Alkyl
	Tyr18	4.97	Hydrophobic	Pi-alkyl
	His32	2.77	Hydrogen bond	Conventional
Pose 2	Asn45	2.59	Hydrogen bond	Conventional
Pose 3	Leu105	2.66	Hydrogen bond	Conventional
Pose 4	Lys108	2.46	Hydrogen bond	Conventional
	Asp97	2.99	Hydrogen bond	Carbon
	His107	3.77	Hydrogen bond	Carbon
	Asn102	2.16	Hydrogen bond	Conventional
Pose 5	Ser112	2.15	Hydrogen bond	Conventional
	Lys114	1.89	Hydrogen bond	Conventional

## 4. Conclusion

The strong binding affinities between non-coated magnetic nanoparticles (MP) or magnetite nanoparticles stabilized with different dopamine ligands (MP_dopamine_) and cellulose (MP_cellulose_) and bovine alpha lactalbumin (BLA) was concluded from the zeta potential analysis, UV-Vis spectroscopy, and steady state fluorescence. Coating the magnetic particles with cellulose and dopamine leads to stronger binding affinities to BLA which is observable from capacity adsorption determination experiment and also can be related to the electrostatic interactions contribution from the salt titration experiment in addition to the hydrogen bonding contribution deduced from the thermodynamic parameters. The involved amino acid residues of BLA in the process of adsorption on the surface of magnetic particles were identified by docking calculation and the Trp-118 was evaluated as key fluorophore. A non-radiative energy transfer with high probability between these particles and BLA was confirmed because of the short distances between MP-sheet and four tryptophan residues in BLA calculated by FRET experiment and PyMol calculations. The cell viability results showed that formation of bioconjugate with BLA causes an increase in the non-toxic range of the considered MP in this project. No altering in the major roles of BLA in transport of the drugs/bioactive compounds is expected because of the no conformal change of the protein in the process of binding to the magnetic nanoparticles. This important characteristic in addition to extremely strong binding affinity makes these particles new model nanostructures for nanomedicine orientated applications.

## Conflicts of interest

There are no conflicts to declare.

## Supplementary Material

RA-010-C9RA09045B-s001
