# Supplementary material for: Interaction of cellulose and nitrodopamine coated superparamagnetic iron oxide nanoparticles with alpha-lactalbumin
Source: RSC Adv. 2020 Mar 6;10(16):9704–16. doi: 10.1039/c9ra09045b (PMC9050155; doi:10.1039/c9ra09045b)
Supplement: RA-010-C9RA09045B-s001 [file RA-010-C9RA09045B-s001.pdf]

Electronic Supplementary Material (ESI) for RSC Advances.
This journal is © The Royal Society of Chemistry 2020

SUPPORTING INFORMATION

Interaction of cellulose coated and nitrodopamine coated superparamagnetic iron oxide nanoparticles with alpha-lactalbumin

Fakhrossadat Mohammadi,[a]* Marzieh Moeeni,[a] Chengnan Li,[b] Rabah Boukherroub[b] and Sabine Szunerits[b]*

[a] *Department of Chemistry, Institute for Advanced Studies in Basic Sciences (IASBS), 444 prof. Sobouti Blvd., Gava Zang, Zanjan 45137-66731, Iran*

[b] *Univ. Lille, CNRS, Centrale Lille, ISEN, Univ. Valenciennes, UMR 8520-IEMN, F-59000 Lille, France*

langmuir Isotherms

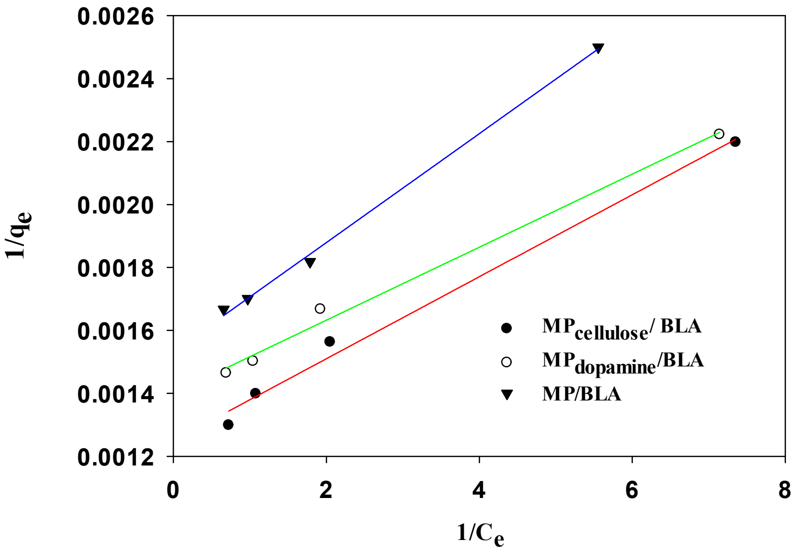

To whom correspondence should be send to: Fakhrossadat Mohammadi (fmohammadi@iasbs.ac.ir; Fax: +98-24-33153232 ; Tel: +98-24-33153218) Dr. Sabine Szunerits (sabine.szunerits@univ-lille1.fr Tél. +33 (0)3 62 53 17 25 Fax. +33 (0)3 62 53 17 01)

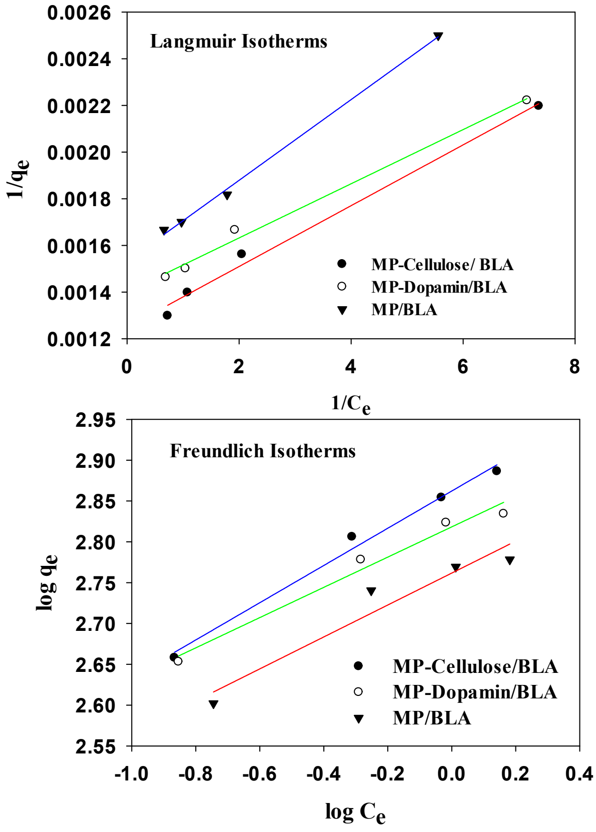

Figure S1. Langmuir and Freundlich isotherm plots for adsorption of BLA on the surface of magnetic particles

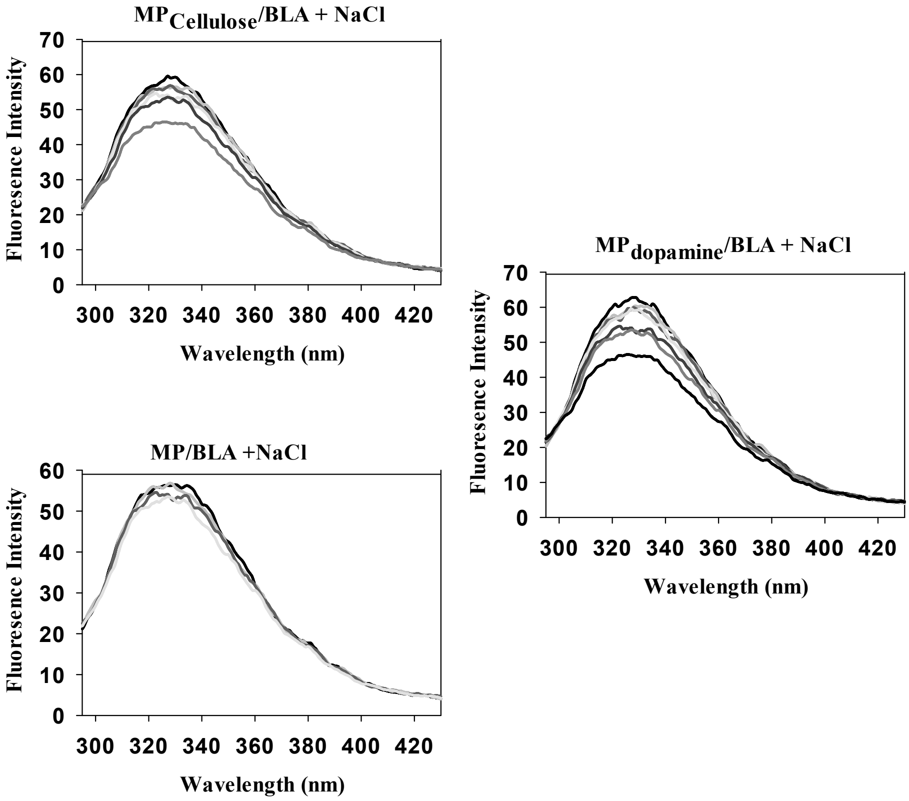

Figure S2: Fluorescence spectra of BLA (2.5 μM) in the presence of magnetic particles with increasing NaCl concentration (0-20 mM).

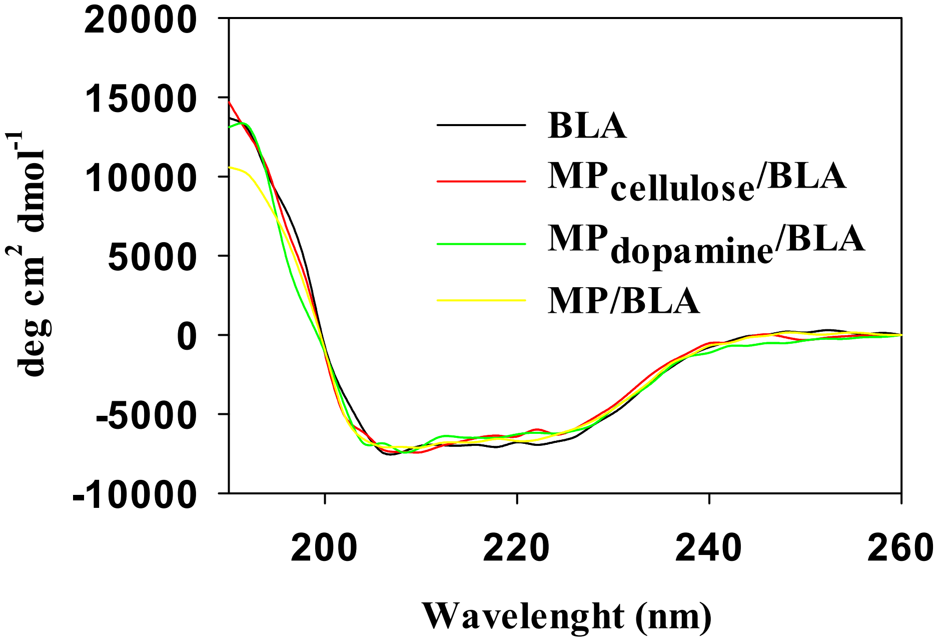

Figure S3: Change in far-UV circular dichroism spectra of BLA in the absence and presence of magnetic particles.

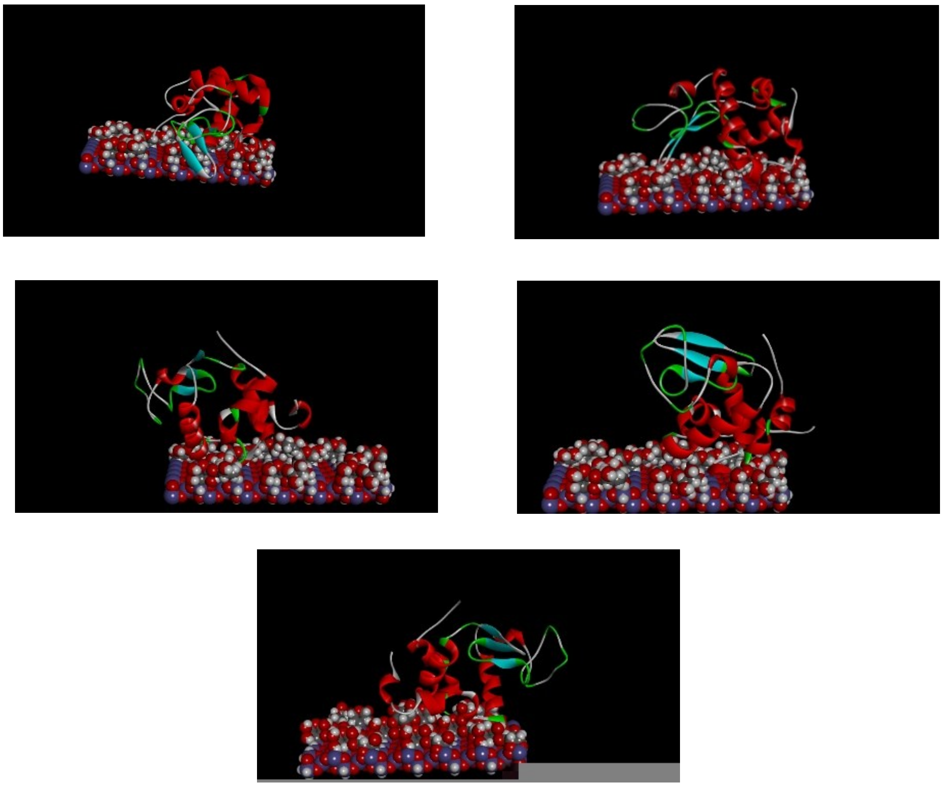

Figure S4: Representation of 5 best docked poses of $MP_{Cellulose}$/BLA interactions

(a)

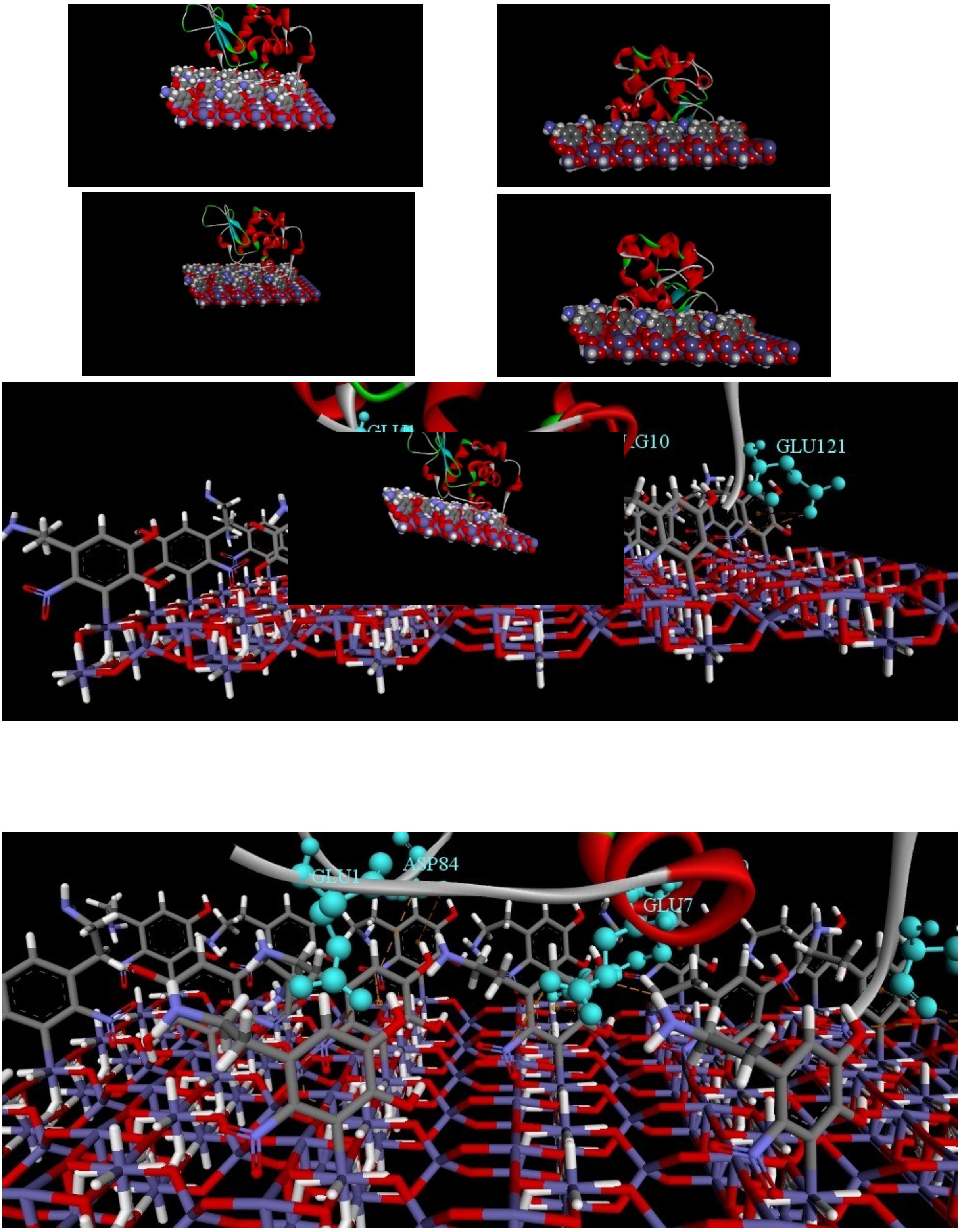

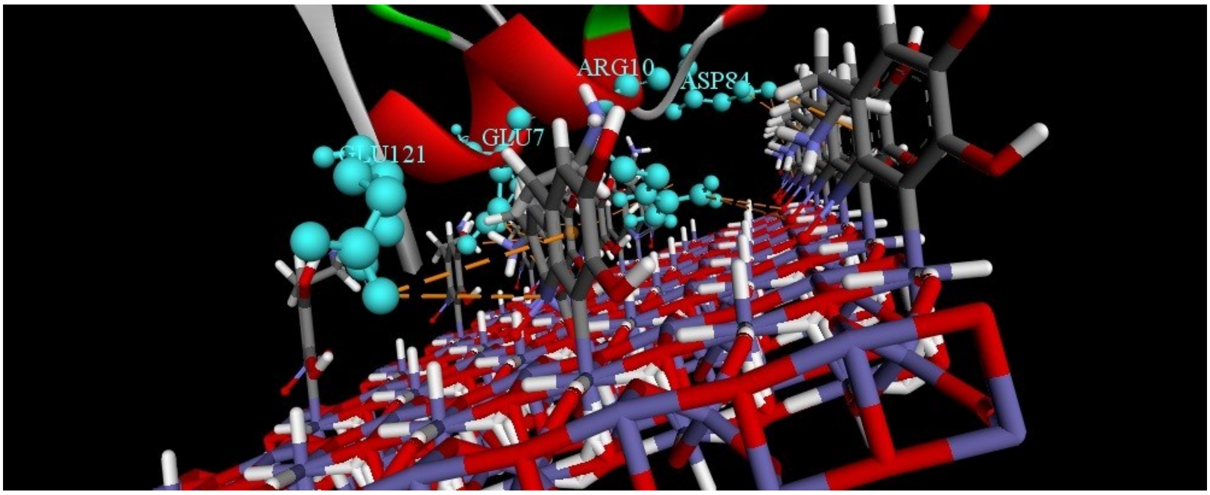

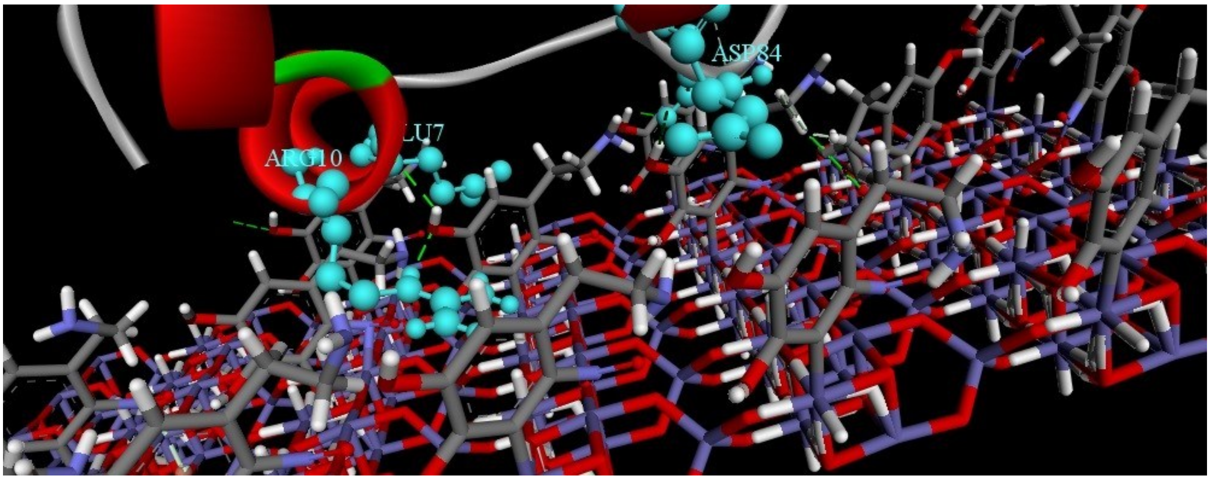

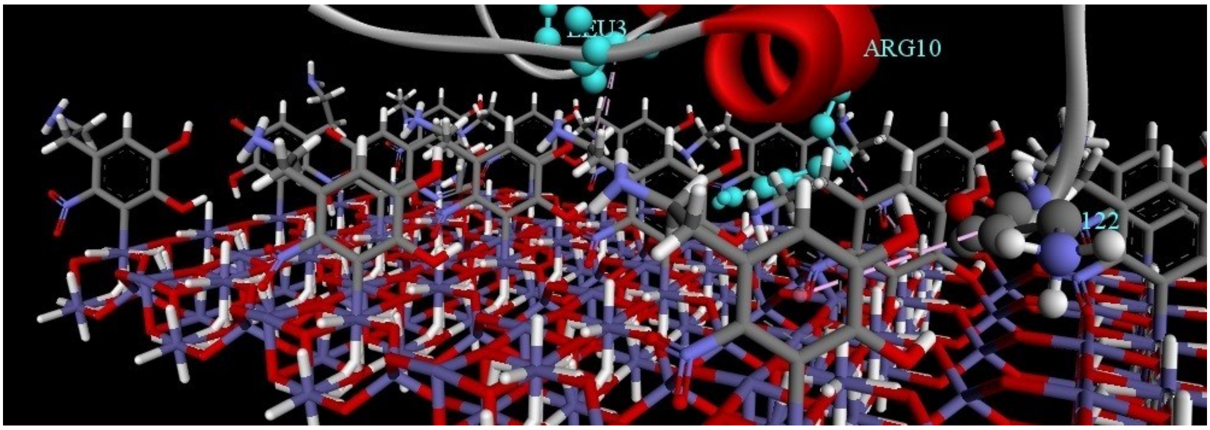

C)

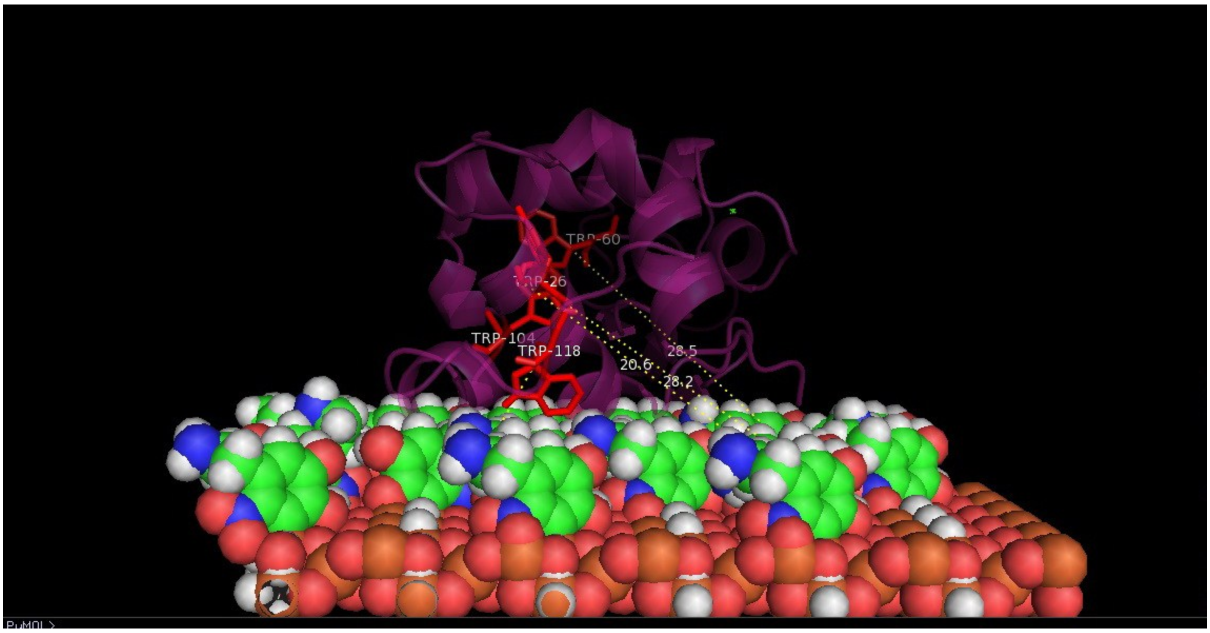

Figure S5: (a) Representation of 5 best docked poses of $MP_{dopamine}$/BLA interactions, b) The interface interactive amino acids and the $MP_{dopamine}$ -Sheet and the hydrogen bindings (in green color), electrostatic bindings (in orange color) and hydrophobic bindings(in pink color) which contributed in the binding site, c) The calculated distances between $MP_{dopamine}$ -sheet and four tryptophans in BLA rendered by pymo

(a)

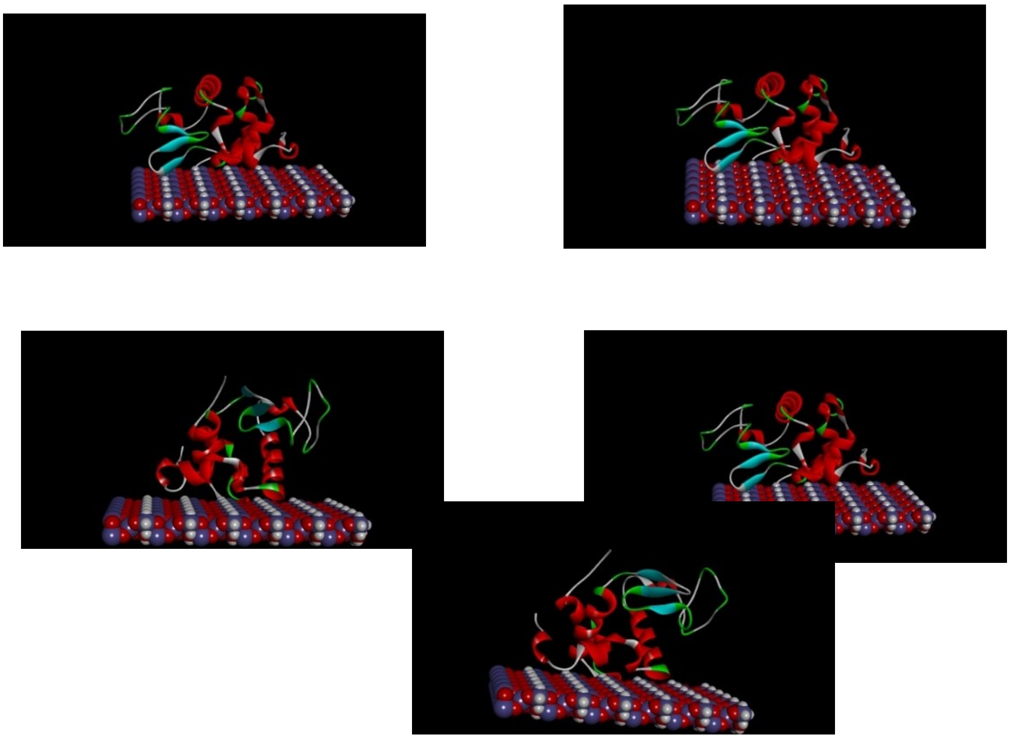

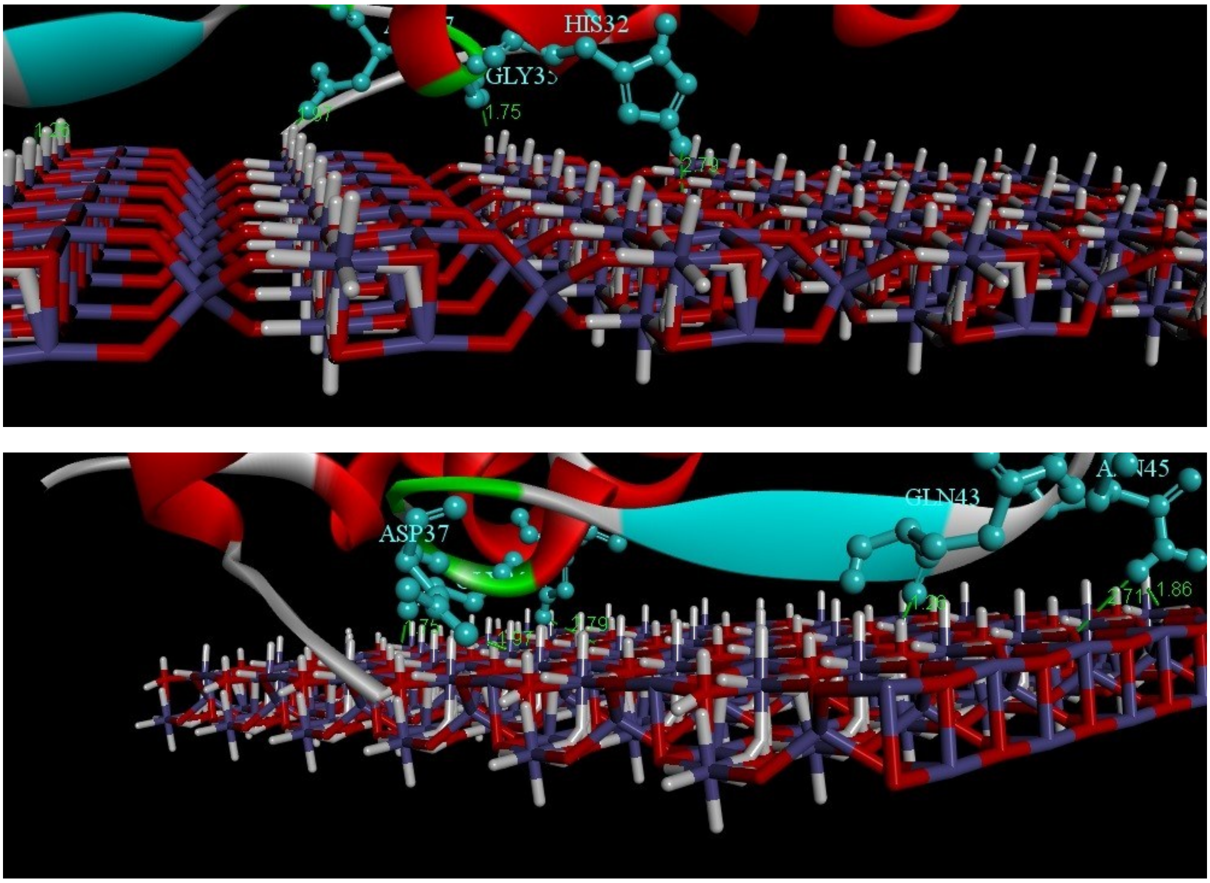

(c)

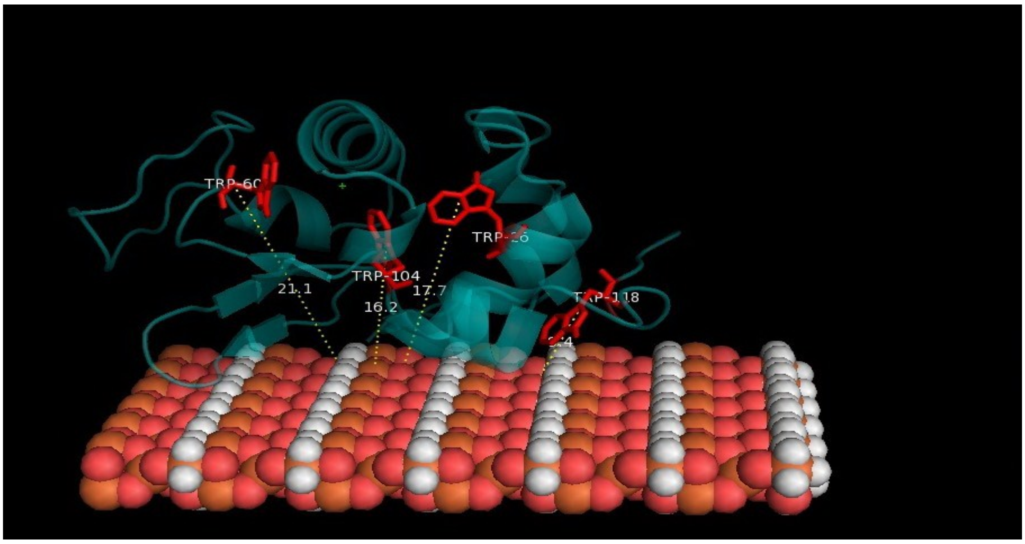

Figure S6: a) Representation of 5 best docked poses of MP/BLA interactions, b) the interface interactive amino acids and the MP-Sheet and the hydrogen bindings (in green color) which contributed in the binding site, c) The calculated distances between MP-sheet and four tryptophans in BLA rendered by pymol

(a)

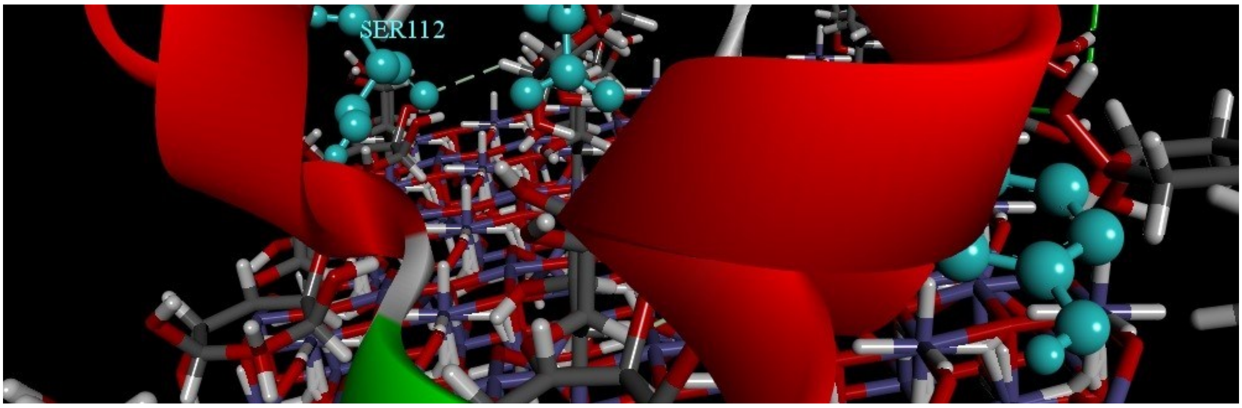

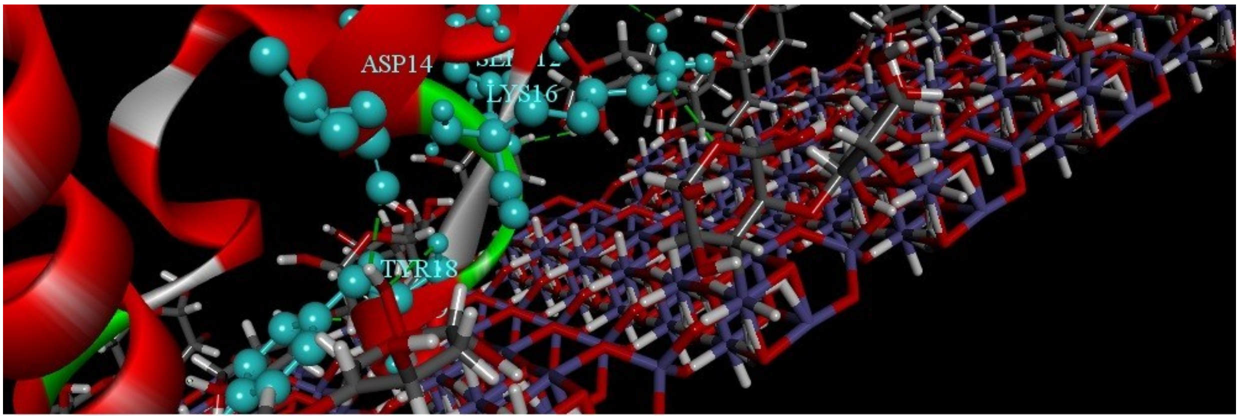

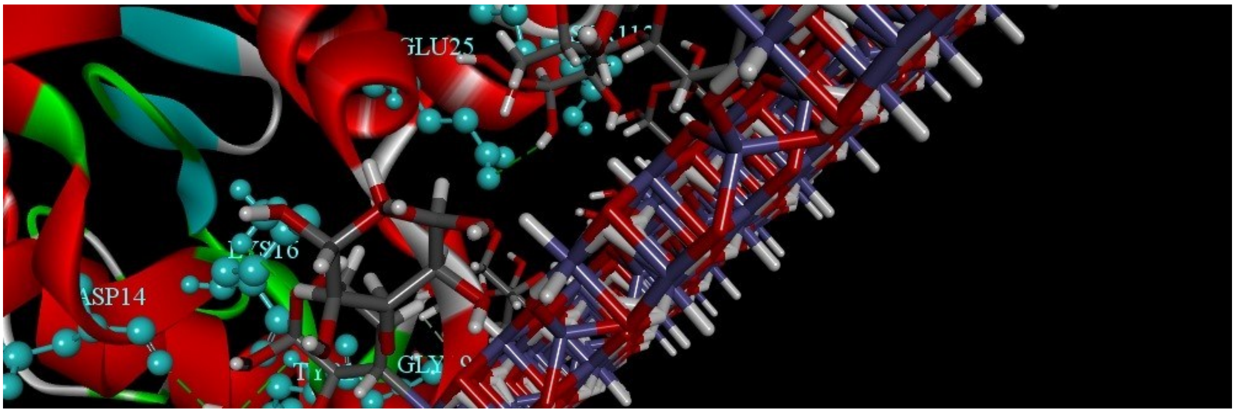

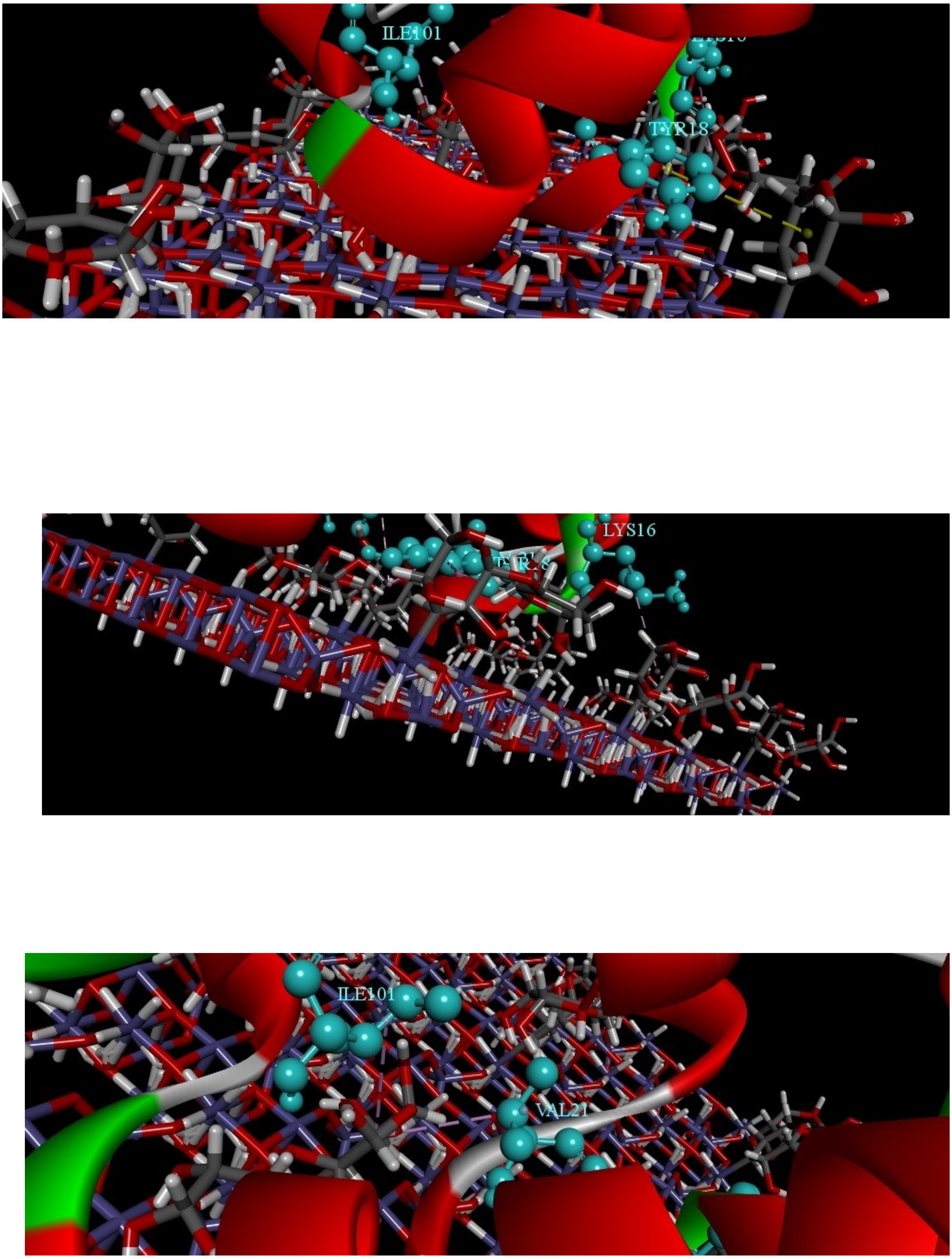

Figure S7. The interface interactive amino acids and the $MP_{cellulose}$-Sheet and the hydrogen bindings (in green color) and hydrophobic binding (in pink color) which contributed in the binding site.

S1 1, Foester Theory

$$E = 1 - \frac{F}{F_0} = \frac{R_0^6}{R_0^6 + r^6} \qquad (8)$$

with $R_0^6 = 8.79 \times 10^{-25}[\kappa^2 n^{-4} \phi J(\lambda)]$ (9)

$$J(\lambda) = \frac{\int_0^{\infty} F_D(\lambda)\varepsilon_A(\lambda)\lambda^4\, d\lambda}{\int_0^{\infty} F_D(\lambda) d\lambda} \qquad (10)$$

where r is the distance between the donor and acceptor (E) R_0 the Förster critical distance where efficiency of the energy transfer between donor and acceptor is 50%, K^2 the relative orientation of the transition dipoles of the donor and acceptor ($K^2 = 2/3$ for a random orientation), n the average refractive index of medium ($n = 1.336$), Φ the fluorescence quantum yield of the donor and J a factor describing the overlapping between the emission spectrum of the donor and the absorption spectrum of the acceptor with $F(\lambda)$ being the fluorescence intensity of the donor at corresponding wavelength λ, $\varepsilon(\lambda)$ the molar absorption coefficient of the acceptor at wavelength λ..

Table S1: The total energy and the interface amino acids of various orientations of MP/BLA interaction.

Docked pose	Total energy (a.u.)	Amino acids
**1**	-481.17	Met1X, GLu1, Gln2, Thr4, Phe31, His32, Thr33, Ser34, Gly35, Tyr36, Asp37, Gln39, Ala40, Ile41, Val42,Gln43, Asn44, Asn45, Thr48, Gln54, Ala109, Lys114, Gln117,**Trp 118**
**2**	-467.10	Met1X, Gln2, Lys5, Phe31, His32, Thr33, Ser34, Gy35, Tyr36, Asp37, Gln39, Ala40, Ile41, Val42,Gln43, Asn44, Asn45, Thr48, Gln54, **Trp118**, Lys122
**3**	-465.69	Phe31, His32, Thr33, Lys114, Ser34, Gy35, Val42,Gln43, Asn44, Asn45, Asp46, Ser47, Glu49, Gln54, Asn56, Ly58,

		Ile59, Trp60, Ser70, Val99, Asn102, Tyr103, Trp104, Leu105, Ala106, Leu110
**4**	-464.55	Tyr18, Gly19, Gly20, Val21, Glu25, Ile95, Leu96, Asp97, Lys98, Val99, Gly100, Ile101, Asn102, His107, Lys108, Cys111, Ser112, Glu113, Lys114
**5**	-462.85	Gly19, Gly20, Val21, Ser22, Glu25Leu96, Leu96, Asp97, Lys98, Val99, Gly100, Ile101, Asn102, His107, Lys108, Cys111, Ser112, Glu113, Lys114, Leu115, Asp116

Table S2: The parameters of the five stable MP/BLA complex, as obtained by molecular docking. The calculated distances between the interface interactive amino acids and the MP-Sheet and the types of bindings which contributed in the binding site

Docked pose	Amino acids	Distances (A°)	Category	Type
**Pose1**	His32	2.79	Hydrogen bond	Conventional
	Asn45	1.86	Hydrogen bond	Conventional
	Asn45	2.70	Hydrogen bond	Conventional
	Asp37	1.97	Hydrogen bond	Conventional
	Gly35	1.75	Hydrogen bond	Conventional
	Gln43	1.26	Hydrogen bond	Conventional
**Pose2**	His32	2.77	Hydrogen bond	Conventional
	Asn45	2.59	Hydrogen bond	Conventional
**Pose3**	Leu105	2.66	Hydrogen bond	Conventional
**Pose4**	Lys108	2.46	Hydrogen bond	Conventional
	Asp97	2.99	Hydrogen bond	Carbon
	His107	3.77	Hydrogen bond	Carbon
**Pose5**	Asn102	2.16	Hydrogen bond	Conventional
	Ser112	2.15	Hydrogen bond	Conventional
	Lys114	1.89	Hydrogen bond	Conventional

Table S3: The total energy and the interface amino acids of various orientations of MP $_{dopamine}$/BLA interaction.

Docked pose	Total energy (a.u.)	Amino acids
**1**	-663.89	Met1X, GLu1, Gln2, Leu3,Thr4, Lys5, Cys6, Glu7, Vl8, Phe9, Arg10, Glu11, Lys13, Thr38, Gln39, Leu81, Asp82, Asp83, Asp84, Thr86, Cys120, Glu121, Lys122
**2**	-649.97	Leu3,Thr4, Lys5, Cys6, Glu7, Vl8, Phe9, Arg10, Glu11, Lys13, Thr38, Ala109, Leu110, Cys111, Ser112, Glu113, Lys114, Asp116, Gln117, **Trp118**
**3**	-584.65	Met1X, GLu1, Gln2,Lys5, Cys28, Phe31, His32, Thr33, Ser34, Gy35, Tyr36, Asp37, Gln39, Ala40, Ile41, Val42, Gln54, Trp104, Leu105, Ala106, His107, Lys108, Ala109, Leu110, Cys111, Ser112, Glu113, Lys114, Asp116, Gln117, **Trp118**
**4**	-574.62	Asp37, Gln2, His32, Met1x, Glu1, Gly35, Ala109, Leu110, Phe31, **Trp118**
**5**	-557.43	Arg10, Lys13, Glu7, Cys6, Lys122, Leu3

Table S4: The parameters of the five stable MP$_{dopamine}$/ BLA complex, as obtained by molecular docking. The calculated distances between the interface interactive amino acids and the MP$_{dopamine}$ Sheet and the types of bindings which contributed in the binding site

Docked pose	Amino acids	Distances (A°)	Category	Type
**Pose1 (the most stable)**	Arg10	5.39	Electrostatic	Attractive charge
	Arg10	4.95	Electrostatic	Attractive charge
	Asp84	5.22	Electrostatic	Attractive charge
	Glu121	4.80	Electrostatic	Attractive charge
	Glu7	3.74	Electrostatic	Attractive charge
	Glu1	4.85	Electrostatic	Attractive charge
	Arg10	2.10	Hydrogen bond	Conventional
	Lys122	2.97	Hydrogen bond	Conventional
	Glu7	2.76	Hydrogen bond	Conventional
	Asp83	2.61	Hydrogen bond	Conventional
	Asp83	2.99	Hydrogen bond	Conventional
	Glu1	1.83	Hydrogen bond	Conventional
	Lys122	3.56	Hydrogen bond	Carbon
	Asp83	2.95	Hydrogen bond	Carbon
	MetX1	2.64	Hydrogen bond	Carbon

	Glu1	2.36	Hydrogen bond	Carbon
	Lys13	2.93	0Hydrogen bond	Pi-Cation; Pi-Donor
	Glu1	4.93	Electrostatic	Pi-Anion
	Glu7	3.95	Electrostatic	Pi-Anion
	Asp83	4.82	Electrostatic	Pi-Anion
	Asp84	4.95	Electrostatic	Pi-Anion
	Glu121	4.74	Electrostatic	Pi-Anion
	Leu3	3.85	Hydrophobic	Alkyl
	Leu3	4.06	Hydrophobic	Alkyl
	Lys122	5.44	Hydrophobic	Pi-Alkyl
	Arg10	4.82	Hydrophobic	Pi-Alkyl
**Pose2**	Asp37	4.96	Electrosta0tic	Attractive charges
	Lys114	1.59	Hydrogen Bond	Conventional
	Asp37	2.90	Hydrogen Bond	Conventional
	His32	1.68	Hydrogen Bond	Conventional
	Leu110	2.90	Hydrogen Bond	Conventional
	Thr33	2.05	Hydrogen Bond	Carbon
	His32	3.94	Electrostatic	Pi-Cation
	Ala109	3.03	Hydrophobic	Pi-Sigma
	Phe31	4.66	Hydrophobic	Pi-Pi Stacked
	His32	4.86	Hydrophobic	Pi-Pi Stacked
	Leu110	4.58	Hydrophobic	Alkyl
	Trp118	4.48	Hydrophobic	Pi-Alkyl
	Ala40	5.32	Hydrophobic	Pi-Alkyl
	Met1x	4.26	Hydrophobic	Pi-Alkyl
